# Transcriptome-Wide Analysis of UTRs in Non-Small Cell Lung Cancer Reveals Cancer-Related Genes with SNV-Induced Changes on RNA Secondary Structure and miRNA Target Sites

**DOI:** 10.1371/journal.pone.0082699

**Published:** 2014-01-08

**Authors:** Radhakrishnan Sabarinathan, Anne Wenzel, Peter Novotny, Xiaojia Tang, Krishna R. Kalari, Jan Gorodkin

**Affiliations:** 1 Center for non-coding RNA in Technology and Health, Section for Animal Genetics, Bioinformatics and Breeding, IKVH, University of Copenhagen, Frederiksberg, Denmark; 2 Bioinformatics Centre, Department of Biology and Biotech Research and Innovation Centre, University of Copenhagen, Copenhagen, Denmark; 3 Division of Biostatistics and Bioinformatics, Department of Health Sciences Research, Mayo Clinic, Rochester, Minnesota, United States of America; 4 Department of Cancer Biology, Mayo Clinic Comprehensive Cancer Center, Jacksonville, Florida, United States of America; Keio University, Japan

## Abstract

Traditional mutation assessment methods generally focus on predicting disruptive changes in protein-coding regions rather than non-coding regulatory regions like untranslated regions (UTRs) of mRNAs. The UTRs, however, are known to have many sequence and structural motifs that can regulate translational and transcriptional efficiency and stability of mRNAs through interaction with RNA-binding proteins and other non-coding RNAs like microRNAs (miRNAs). In a recent study, transcriptomes of tumor cells harboring mutant and wild-type *KRAS* (V-Ki-ras2 Kirsten rat sarcoma viral oncogene homolog) genes in patients with non-small cell lung cancer (NSCLC) have been sequenced to identify single nucleotide variations (SNVs). About 40% of the total SNVs (73,717) identified were mapped to UTRs, but omitted in the previous analysis. To meet this obvious demand for analysis of the UTRs, we designed a comprehensive pipeline to predict the effect of SNVs on two major regulatory elements, secondary structure and miRNA target sites. Out of 29,290 SNVs in 6462 genes, we predict 472 SNVs (in 408 genes) affecting local RNA secondary structure, 490 SNVs (in 447 genes) affecting miRNA target sites and 48 that do both. Together these disruptive SNVs were present in 803 different genes, out of which 188 (23.4%) were previously known to be cancer-associated. Notably, this ratio is significantly higher (one-sided Fisher's exact test p-value = 0.032) than the ratio (20.8%) of known cancer-associated genes (n = 1347) in our initial data set (n = 6462). Network analysis shows that the genes harboring disruptive SNVs were involved in molecular mechanisms of cancer, and the signaling pathways of LPS-stimulated MAPK, IL-6, iNOS, EIF2 and mTOR. In conclusion, we have found hundreds of SNVs which are highly disruptive with respect to changes in the secondary structure and miRNA target sites within UTRs. These changes hold the potential to alter the expression of known cancer genes or genes linked to cancer-associated pathways.

## Introduction

Next-generation genome sequencing is now widely used for the identification of genetic variations in cancer genomes [1], [2]. Non-small cell lung cancer (NSCLC) is the most common form of lung cancer and it is often found with activating mutations in the *KRAS* oncogene which causes the tumor cells to be aggressive and resistant to chemotherapy [3]–[5]. In a recent study, Kalari et al. [6] performed transcriptome-wide sequencing of NSCLC and identified differentially expressed genes, alternate splicing isoforms and single nucleotide variants (SNV) for tumors with and without *KRAS* mutations. A network analysis was performed with the genes showing differential expression (374 genes), alternate splicing (259 genes) and SNV-related changes (65 genes) that are differentially present in lung tumor groups with and without *KRAS* mutations. Integrated pathway analysis identified NFκB, ERK1/2 and AKT pathways as the most significant pathways differentially deregulated in *KRAS* wild-type as compared with *KRAS* mutated samples.

A single nucleotide variant (SNV) is a nucleotide change at a single base position that occurs at a low frequency (also referred as a rare variant). SNVs observed in tumor cells are mostly somatic variants and very few are germ-line variants. Genome-wide association studies (GWAS) report that SNVs mostly occur in non-coding regions compared to coding (exonic) regions of RNAs [7]. In the past, however, most studies have been focused on the effect of SNVs in coding regions (known as cSNVs or nsSNVs) [8] rather than the effect of SNVs in the regulatory non-coding DNA or non-coding RNA (rSNV). In the case of NSCLC, Kalari et al. [6] identified a total of 73,717 unique SNVs present in and around (+/−5 kb) RefSeq genes. Of these, 23,987 were cSNVs and their effects on coding regions have previously been predicted (see [6] for more details). The effects of rSNVs that are located in untranslated regions (UTRs) of protein-coding genes, however, need to be analyzed.

It is well known that UTRs play crucial roles in post-transcriptional regulation including mRNA stability [9], transport [10], localization [11], [12], translational activation [13] and repression [14], [15]. These functional regulations are carried out by *cis*-regulatory elements present in 5′ and 3′ UTRs. Notably, some of the *cis*-regulatory elements are structured, e.g., iron-responsive element (IRE), internal ribosome entry site (IRES) and selenocysteine insertion sequence (SECIS). The primary structure of *cis*-regulatory elements is also important for the binding of *trans*-acting RNA-binding proteins or other non-coding RNAs. For example, microRNAs (miRNAs) are small non-coding RNAs (about 22 nt) that bind to target sites mostly present in 3′ UTRs. This interaction results in either the cleavage of target mRNAs or repression of their translation. Several studies have reported that miRNA-mediated gene regulation plays a major role in cancer cells and such regulation has been considered as a potential drug target (see review [16]). All this evidence supports both sequence and structural motifs of UTRs being important for the control of gene expression.

The occurrence of genetic variation(s) in UTRs could potentially affect their sequence and/or structural motifs and thus lead to changes in post-transcriptional regulation [17]–[20]. For example, a SNP in a let-7 miRNA target site (miRTS) in the 3′ UTR of *KRAS* has been identified to affect the binding of let-7 miRNAs. This results in the overexpression of *KRAS*, leading to the increased risk of NSCLC [21]. In addition, recent studies report that genetic variation can potentially create, change or destroy miRNA targets sites, which results in dysregulation of the target mRNA [22], [23]. Notably this has been identified in tumor cells as well [24]. Furthermore, a cancer-driven mutation present in an IRES in human *p53* mRNA alters the structure of the IRES element, which inhibits binding of a trans-acting factor essential for translation [25]. The recent number of web servers and data bases developed to deal with variants affecting miRTSs also demonstrates the growing importance of target site variants [22], [26]–[28].

In this study, we predict the possible effects of 29,290 SNVs associated with NSCLC that are located in the UTR regions of mRNAs. The local effect of SNVs on the secondary structure of UTRs is predicted using RNAsnp [29] and the effect of SNVs on miRTSs in the UTR is predicted using TargetScan [30] and miRanda [31], which were shown to be among the more reliable miRNA target prediction methods [32]. The experimentally identified miRNA-mRNA maps, using Argonaute (Ago) cross-linking immunoprecipitation coupled with high-throughput sequencing (CLIP-Seq), are further used to reduce the false positive predictions of miRTSs [33].

## Materials and Methods

### Data sources

The SNVs identified through RNA-sequencing of 15 primary lung adenocarcinoma tumors (8 with *KRAS* mutation and 7 without *KRAS* mutation) were extracted from Kalari et al. [6]. It should be noted that the previous study [6] had no RNA-sequencing data on normal cells so it was not possible to separate SNVs derived from the germ-line or somatic mutation. Thus, the data set obtained, 29,290 UTR SNVs in 6462 coding genes, derived from both germ-line and somatic variants that were expressed in lung adenocarcinoma tumors. Based on the overlap of these SNVs with the dbSNP (135 build), we could estimate that 40% of the 29,290 SNVs are germ-line variants. For those SNVs that overlap with dbSNP entries, we also extracted the SNPs in linkage dis-equilibrium using the SNAP server [34] (version 2.2; with the default parameters: r^2^≥0.8, distance limit 500 kb, SNP data set 1000 Genomes pilot 1, and population panel CEU).

**Figure 1:**
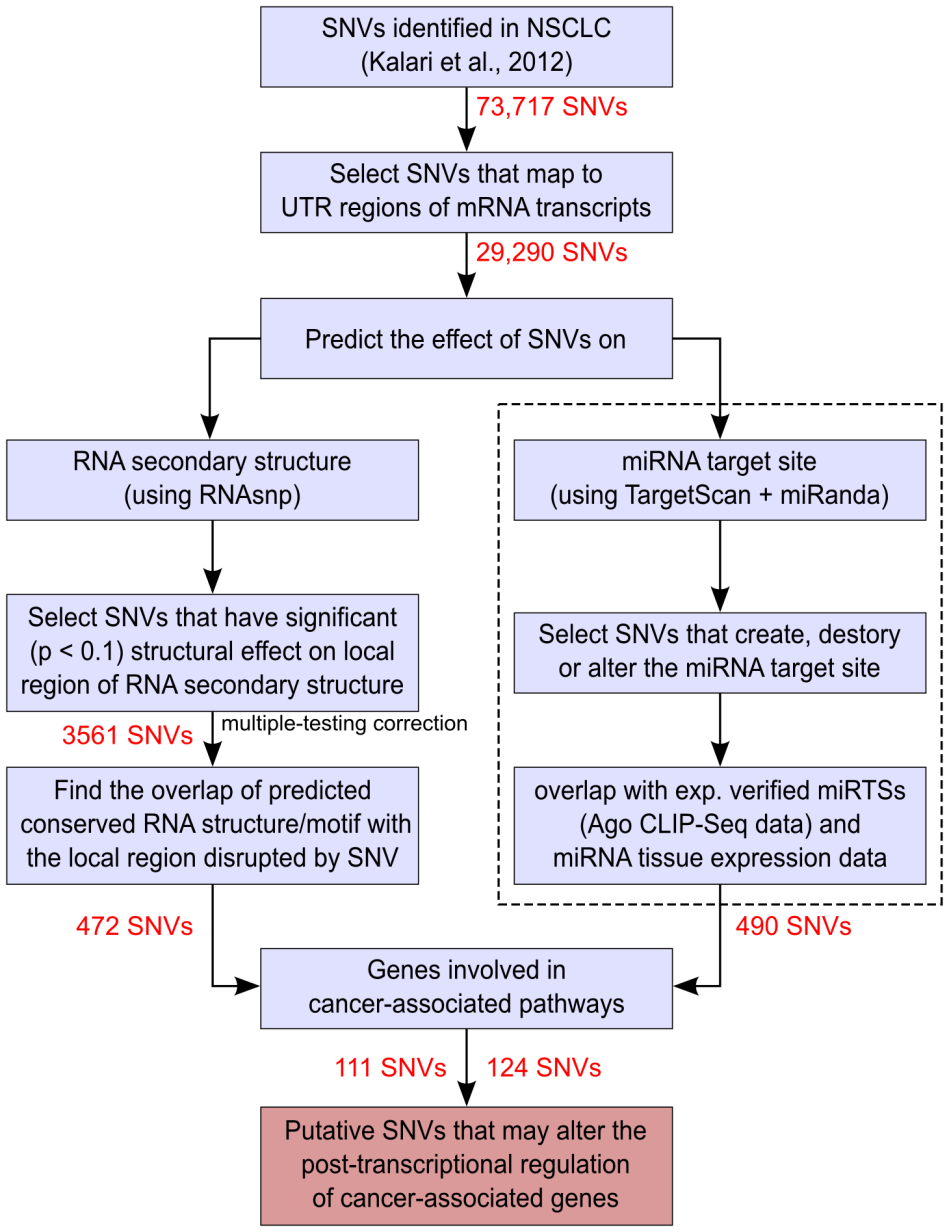
Pipeline for the analysis of effect of SNVs on UTRs of mRNA.

RefSeq mRNA sequences corresponding to the 6462 genes (hg19 Build) were downloaded from the UCSC genome browser (http://genome.ucsc.edu) [35]. For genes with multiple transcripts, all isoforms were considered. By mapping of 29,290 SNVs to these RefSeq mRNA sequences, we obtained 3646 in 5′ UTRs, 25,627 in 3′ UTRs and 17 in both 5′ and 3′ UTR of overlapping transcripts. These SNVs were further subjected to our comprehensive pipeline (Figure 1) to predict their effect on RNA secondary structure and miRNA target sites, which is described in the following sections.

A list of cancer-associated genes was obtained from COSMIC [36] and Qiagen/SABioSciences [37]. This list includes 1347 of the 6462 genes considered in this analysis. In order to find the enrichment of genes carrying disruptive SNVs, which have effect on secondary structure and/or miRTSs, in cancer, we performed a one-sided Fisher's exact test. This was computed from a 2×2 contingency table (n = 6462) with the number of genes carrying/not carrying disruptive SNVs on the one side and the number of cancer-associated/other genes on the other side. Similarly, the enrichment for disruptive SNVs in cancer-associated genes was computed by classifying the total number of SNVs (n = 29,290) into disruptive/non-disruptive SNVs on the one hand, and those being and not being present in cancer-associated genes on the other.

A set of experimentally verified examples of SNPs with effects on miRNA target sites has been extracted from the literature (see Table S1). These 19 SNPs (affecting 25 miRNA-mRNA interactions) have been used to test the filtration criteria used in the miRNA part of our pipeline.

### Prediction of SNVs' effect on RNA secondary structure

The effect of SNVs on RNA secondary structure was predicted using RNAsnp (version 1.1) [29]. The wild-type mRNA sequences and the SNVs were given as input along with default parameters of RNAsnp. For each SNV, RNAsnp considered a window of +/−200 nts around the SNV position to generate the wild-type (WT) and mutant (MT) subsequences and computed their respective base pair probability matrices 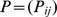 and 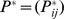. Then, the difference between the base pair probability of wild-type and mutant structure was measured using Euclidean distance (d) and Pearson correlation coefficient (r) for all local regions 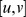 within the subsequence. For completeness, we briefly summarize these two measures as follows. The first one computes the difference between two matrices directly by
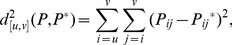
(1)where 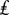 is the probability of bases *i* and *j* being paired. The second measure uses the position-wise pair probabilities 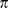. For a local region 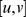, the vector 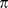 contains the elements 
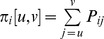
. Then the difference between two vectors 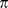 and 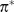 is measured by
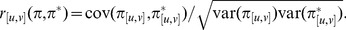
(2)


Finally, a local region predicted with maximum Euclidean distance (d_max_) or minimum Pearson correlation coefficient (r_min_) and the corresponding p-value is then reported. We employ both measures independently as both measures hold their respective strengths and weaknesses (see [29] for details). We generated two lists (each with p<0.1) of candidates, d_max_ and r_min_, and each of them is subjected to a multiple-testing correction using the Benjamini-Hochberg procedure [38], which limits the false discovery rate to be no more than a chosen threshold (typically 10%).

To analyze whether the RNAsnp predicted local region is structurally conserved, we used the annotations of conserved RNA secondary structure predictions from our in-house pipeline [39], which makes use of a range of tools including CMfinder [40] and RNAz [41] programs.

### Predicting of SNVs' effect on microRNA target sites

For each SNV in the data set, a subsequence of 30 nts on either side of the SNV position was retrieved. Further, all 2042 human mature miRNA sequences from miRBase (v19) [42] were used to scan for possible target sites in wild-type and mutant (with SNV) subsequences. As a first step, TargetScan (version 6.0) [30] was used to identify pairs of SNVs and miRNAs for which the type of seed match differs between wild-type and mutant or is only present in either of them. The different seed types used by TargetScan are 7mer-1a, 7mer-m8, and 8mer-1a (in increasing strength), where ‘1a’ refers to an adenosine in the miRTS 3′ to the seed match (i.e., opposite the first nucleotide of the miRNA) and ‘-m8’ refers to a Watson-Crick-matched nucleotide in position 8. Subsequently, the interaction energy of these pairs was computed using miRanda (version 3.3a) [31]. As a seed match change is already required by the TargetScan filtration, the parameters for miRanda were set to not weigh the seed region too high (‘-scale 2’ instead of default 4) and with relaxed cutoffs (score 45, energy −5 kcal/mol), in order to capture cases where a poor seed match can be compensated. To classify an interaction as working we later apply a more conservative energy threshold of −11 kcal/mol based on our previous study [43]. For each pair of miRNA and 61mer, only the strongest binding site (lowest 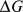) that differs between WT and SNV sequence is retained. Putative interactions are classified as *created*, *destroyed* or *altered* upon mutation based on miRanda predictions. The *create* set contains target sites that are induced by the SNV, i.e., have an interaction energy of −11 kcal/mol or lower in the SNV variant, while no interaction is predicted in the wild-type (either due to score or energy threshold). Similarly, a loss of target site would be recorded in the *destroy* set, if the interaction is predicted for the wild-type but not with the SNV. Finally, the *alter* set contains putative interactions that are predicted with a binding energy of at most −11 kcal/mol for at least one variant. For these, the energy difference observed for the binding of miRNA before and after SNV introduction was measured as their log-ratio *lr* = ld (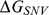/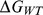), for 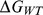
<0. The *lr* is 0 if there is no change in energy; negative if the wild type has the stronger interaction (lower energy), positive otherwise. Given the size of the data set, we focus on the (top) candidates whose absolute *lr* value is above the mean (μ) of absolute *lr* values from all pairs classified as alter. The efficiency of this threshold clearly varies with the data, but it will always retain the top candidates with highest relative energy difference. Even though it should not be seen as a fixed cut-off, we applied it to our set of known examples, where 14 out of 23 interactions exceed the value we applied here (see Table S1). Threshold values based on the distribution of MFE changes have been used in a similar way before [24].

In order to reduce false positive predictions, the miRNA target sites predicted for the wild-type (*destroy* or *alter*) were cross-checked with experimentally identified microRNA-target interaction maps. Those data, derived through Ago CLIP-Seq, was downloaded from starBase [44]. Only SNVs that are located inside stringent Ago CLIP-Seq peak clusters with a biological complexity (BC) of at least two were retained. This filter cannot be used for the interactions from the *create* set, as CLIP-Seq data is available for the wild-type only.

**Figure 2:**
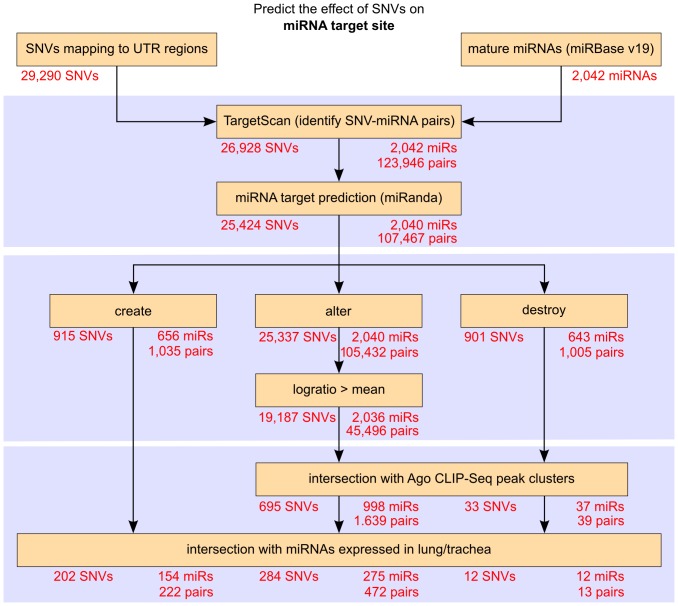
Pipeline for the analysis of SNVs' effect on miRNA target sites in more detail (dashed box from Figure 1). The flow chart shows the different steps of prediction and filtration with the number of individual SNVs, miRNAs, and pairs of these at each stage.

Finally, the set of miRNAs was filtered for those expressed in the respiratory system (lung and trachea) according to the miRNA body map [45]. The overview of the miRNA analysis is described in Figure 2.

From the PhenomiR [46] database, we retrieved information about miRNAs that have been found to be up- or down regulated in lung cancer. This set comprises 264 individual miRNA stem-loop accessions, 3 of which are ‘dead entries’. The remaining 261 stem-loops give rise to 430 mature miRNA products, which we refer to as *lung cancer-associated miRNAs*. In this data set, 27 miRNAs are specific to NSCLC [47] type according to miRNA body map [45]. The later data set is referred as *NSCLC-associated miRNAs*.

### Ingenuity Pathways Analysis

Interactome networks of candidate genes were constructed using Ingenuity Pathway Analysis (IPA) software (Ingenuity® Systems, www.ingenuity.com; build version: 220217; content version: 16542223). Network generation is based on the ‘Global Molecular Network’ in IPA, which comprises an extensive, manually curated set of gene-gene relationships based on findings from the scientific literature. The genes of interest (candidates put out by our pipeline and used as input for IPA) that are also present in this global network are the so-called focus genes. Highly-interconnected focus genes are the starting points in network generation. Additional non-focus genes from the Global Molecular Network might be used as linker genes between small networks. Networks are extended until an approximate size of 35 genes, which is considered optimal for visualization and interpretation (for details see [48]). The p-value computed for each network represents the probability to find the same (or higher) number of focus genes in a randomly selected set of genes from the global network. It is computed by a right-tailed Fisher Exact Test with (non-)focus molecules on the one side and molecules (not) in the network on the other side of a 2×2 contingency table. This is transformed into a score which is the negative log of the p-value. Furthermore, IPA was used to identify the top diseases and disorders, molecular and cellular functions, and canonical pathways associated with the genes in our candidate sets (so called ‘focus genes’). The p-value for a given (disease, function or pathway) annotation describes the likelihood that the association between the input gene set and the annotation is due to random chance. This is also based on a right-tailed Fisher's exact test as specified above.

## Results

### Effect of SNVs on RNA secondary structure

The structural effects of 29,290 UTR SNVs were predicted using RNAsnp (v1.1). Both the Euclidean distance (d_max_) and Pearson Correlation Coefficient (r_min_) measures of RNAsnp (mode 1) were independently employed to predict the effect of SNVs on local RNA secondary structure. The distribution of p-values calculated for the 29,290 UTR SNVs is shown in Figure S1A. At a significance level of 0.1 (chosen from our previous study [29]), 3237 and 3062 SNVs were predicted, respectively, by d_max_ and r_min_ measures. Further, the adjustment for multiple comparisons (using Benjamini–Hochberg procedure [38]) provided 3204 and 1813 SNVs respective to d_max_ and r_min_ measures. After fusing these two lists, we got 3561 unique SNVs in 2411 genes.

Further, we calculated the distance between the location of these 3561 SNVs and the predicted local region where the maximum structural change was detected (Figure S1B). It shows that the majority of the SNVs cause structural change in and around the SNV position. In addition, the length distribution of the predicted local region shows that the majority of SNVs have effect on the local region of size 50 to 100 nts, however, certain SNVs (n = 47) have effect on a global structure where the size of predicted local region exceeds 300 nts (Figure S1C). Furthermore, we checked whether these disruptive SNVs are enriched in GC or AU rich regions, as sequences with such biased nucleotide content have been shown more sensitive to structural changes caused by mutations [49]. For each SNV we computed the GC content of its flanking regions (as previously using 200 nts up- and down- stream), see Materials and Methods. This showed that both the data set SNVs and the disruptive SNVs are highly enriched in the regions with GC content ranging from 40 to 60 percent (see Figure S2), which should therefore make them less sensitive to variations. In addition, we found that there were no significant differences between GC content distributions of disruptive SNVs and the data set SNVs (see Figure S2 with Kolmogorov-Smirnov).

It is known that the UTRs of mRNAs harbor evolutionarily conserved regulatory elements ([50]–[52], see also reviews [53], [54]). Thus, we cross-checked for the overlap between the disrupted local region predicted by RNAsnp and the conserved RNA secondary structures predicted using our in-house pipeline [39] (see Material and Methods sections for details). Interestingly, the local region predicted for 472 SNVs (p-value<0.1) overlap with the predicted conserved RNA secondary structures. These 472 SNVs correspond to 408 genes; out of which 111 SNVs correspond to 98 genes that are involved in cancer-associated pathways (see File S1.xlsx).

Based on the p-value, the above 111 SNVs were further classified into two groups: 28 as high-confidence for which both d_max_ and r_min_ p-value<0.05 (Table 1), and the other 83 as medium-confidence (either d_max_ or r_min_ p-value<0.1) (see File S2.xlsx). We predict that the SNV-induced structural changes in the UTR regions could potentially affect the stability of the mRNA or disrupt the function of regulatory elements present in the UTRs. For example, the SNV A2304C (Table 1) present in the 3′ UTR of *MAPK14* mRNA shows a significant structural change (p-value: 0.0076) in the local RNA secondary structure which is structurally conserved according to both CMfinder and RNAz predictions from our in-house pipeline [39]. This structural conservation shows that the region is under evolutionary pressure to maintain the structure which is likely to have some functional importance. The protein encoded by *MAPK14* gene is a member of the MAP kinase family, which is known to be involved in many pathways related to cell division, maturation and differentiation (reviewed in [55]). Also, it has been predicted to be one of the key players in the lung cancer interactome [6]. Thus the alteration in the gene expression of *MAPK14* at a post-transcriptional level due to the SNV-induced structural change could potentially affect the MAKP14-related signaling pathways.

**Table 1:** List of 28 high-confidence SNVs with p-value<0.05 predicted by both d_max_ and r_min_ measures of RNAsnp.

Gene	mRNA	UTR	SNV	RNAsnp d_max_ (p-value)	% overlap with conserved secondary structure^a^	RNAsnp r_min_ (p-value)	% overlap with conserved secondary structure	dbSNP 135
GSR	NM_001195102	3	A2638G	0.0030	89^$^	0.0213	100^$^	rs1138092
MEF2A	NM_005587	3	A2046U	0.0032	100^$^	0.0238	100^$^	
PPM1A	NM_177952	3	G2231A	0.0059	100^#^	0.0118	100^#^	
MAPK14	NM_139012	3	A2304C	0.0076	100^#^;96^$^	0.0224	91^#^;91^$^	
PHC2	NM_198040	3	A3730C	0.0105	-	0.0137	75^#^	
BECN1	NM_003766	3	U1970C	0.0120	100^#^	0.0487	100^#^	
NFKBIE	NM_004556	3	G1659C	0.0144	84^$^	0.0473	100^$^	
MAPK1	NM_002745	3	U2360G	0.0148	100^#^	0.0262	100^#^	rs13058
DHCR24	NM_014762	3	A4192C	0.0159	100^#^	0.0494	100^#^	
ADAMTS1	NM_006988	3	U4320G	0.0166	94^#^	0.0215	83^#^	
SRF	NM_003131	3	C3504U	0.0173	100^#^	0.0074	100^#^	rs3734681
CASP2	NM_032982	3	U2139C	0.0189	56^#^	0.0230	62^#^	
LFNG	NM_001040167	3	C1838G	0.0215	64^#^	0.0333	-	rs4721752
SH3PXD2A	NM_014631	3	C8560U	0.0223	100^$^	0.0073	-	
KITLG	NM_000899	3	U1057G	0.0226	50^$^;84^#^	0.0080	90^#^	
PRKAB1	NM_006253	3	U1875C	0.0237	100^$^	0.0462	100^$^	
TFG	NM_001195479	5	G309C	0.0256	-	0.0398	56^#^	
FTH1	NM_002032	3	U819G	0.0262	93^#^	0.0259	100^#^	
BCL2L2	NM_001199839	3	C2469A	0.0275	78^#^	0.0294	100^#^	rs3210043
CDKN1C	NM_000076	3	G1334C	0.0290	87^#^	0.0253	87^#^	
TIA1	NM_022173	3	U4082A	0.0316	100^#^	0.0363	100^#^	
NFKBIE	NM_004556	3	U1644G	0.0333	98^#^	0.0347	100^#^	
DAPK3	NM_001348	3	G1662U	0.0334	54^#^	0.0216	94^#^	rs3745982
NCOA1	NM_003743	3	C4893G	0.0342	100^#^	0.0176	100^#^	rs17737058
PCBP4	NM_001174100	3	C1790G	0.0413	59^#^	0.0187	-	
SH3PXD2A	NM_014631	3	U8562A	0.0451	100^$^	0.0096	100^$^	
ID2	NM_002166	5	C143G	0.0464	87^#^	0.0474	83^#^	
GPX3	NM_002084	3	U1552G	0.0474	73^#^	0.0427	100^#^	

^a^ The conserved RNA secondary structure predicted by CMfinder and RNAz programs (through our in-house pipeline [39]) are highlighted with the symbols ^#^ and ^$^, respectively.

**Figure 3:**
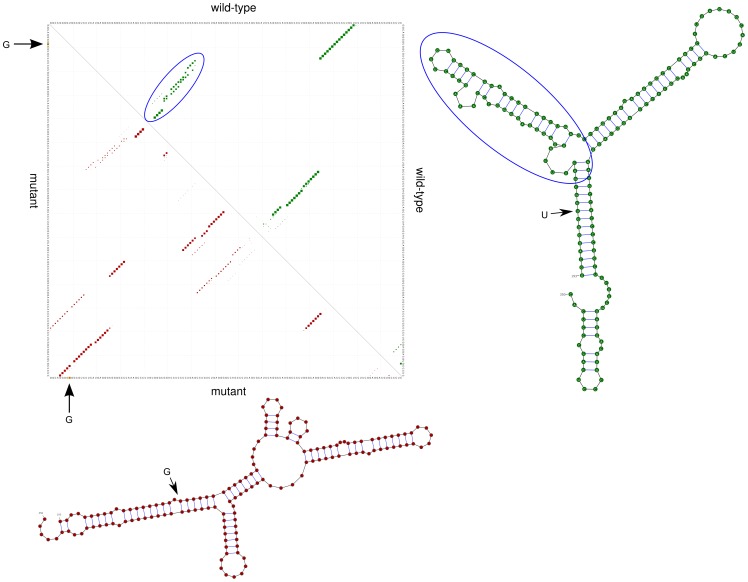
Results of SNV U1552G predicted to cause significant local secondary structure changes in 3′ UTR of GPX3 mRNA. The dot plot from RNAsnp web server [67] shows the base pair probabilities corresponds to the local region predicted with significant difference (*d_max_* p-value: 0.0474) between wild-type and mutant. The upper triangle represents the base pair probabilities for the wild-type (green) and the lower triangle for the mutant (red). On the sides, the minimum free energy (MFE) structure of the wild-type and mutants are displayed in planar graphic representation. The SECIS region is highlighted in blue circle and the SNV position is indicated with arrow mark.

As another example, the gene *GPX3* is responsible for the coding of plasma glutathione peroxidase, an antioxidant enzyme that contains selenocysteine in its active site and catalyzes the reduction of hydrogen peroxide. The amino acid selenocysteine is encoded by the UGA codon, which normally functions as a stop codon. In the *GPX3* mRNA, the alternate recognition of a UGA codon as a selenocysteine codon is mediated by the *cis*-acting regulatory element, selenocysteine insertion sequence (SECIS), present in the 3′ UTR and other *trans*-acting co-factors [56]. The SNV U1552G (Table 1) located in the 3′ UTR of *GPX3* mRNA was predicted to cause significant structural effect (p-value: 0.0474) in the local region which contains the SECIS regulatory element. Figure 3 shows the base pair probabilities corresponding to the local region (NM_002084:1544 to 1692) of wild-type and mutant mRNA. It can be seen that the wild-type has higher base pair probabilities to form the stable stem-loop structure of SECIS (highlighted with a circle in Figure 3), whereas in the mutant form it is disrupted due to the SNV, which is located outside the SECIS region. Previous study has shown that the characteristic stem-loop structure of SECIS is essential for the efficiency of UGA recoding *in vivo* and *in vitro* [56]. Based on this, we speculate that the SNV U1552G induced structural change in the SECIS element may affect the efficiency of UGA recoding.

Further, considering both the set of high-confidence and medium-confidence SNVs, we found that the genes (n = 15) listed in Table 2 harbor more than one disruptive SNV in the predicted conserved structural region of mRNA. For example, the gene *ID2* encodes for DNA-binding protein inhibitor ID-2, which is a critical factor for cell proliferation and differentiation in normal vertebrate development. Overexpression of the ID-2 protein is frequently observed in various human tumors, including NSCLC [57]. In the mRNA sequence of *ID2* gene, two SNVs located in the 5′ UTR region were independently predicted to cause significant local structural change in the conserved region. Previous studies have shown that SNP or mutation induced structural changes in the 5′ UTRs can lead to uncontrolled translation or overexpression of the respective proteins [58], [59]. We predict that the two SNVs that cause significant change in the structurally conserved region could affect the translation efficiency of *ID2* mRNA.

**Table 2:** List of genes which have more than one disruptive SNV (combined high-confidence and medium-confidence candidates) in the UTRs.

Gene	mRNA	UTR	SNV^a^	RNAsnp (*p*-value)^b^	% overlap of predicted local region with conserved RNA secondary structure^c^	dbSNP 135
SH3PXD2A	NM_014631	3	C8560U	0.0223	100^$^	
SH3PXD2A	NM_014631	3	U8562A†	0.0451	100^$^	
MAPK1	NM_002745	3	G1633A	0.0815	100^#^	rs41282607
MAPK1	NM_002745	3	U2360G†	0.0148	100^#^	rs13058
ACOX1	NM_004035	3	U4708G	0.0650	100^#^	
ACOX1	NM_004035	3	A6386U	0.0750	59^#^	
ADAMTS1	NM_006988	3	U4320G	0.0166	94^#^	
ADAMTS1	NM_006988	3	U3449C	0.0626	86^$^	
CDC42	NM_001039802	5	C159A	0.0665	100^$^	
CDC42	NM_001039802	5	G152A	0.0901	100^$^	
ID2	NM_002166	5	C143G†	0.0464	87^#^	
ID2	NM_002166	5	C129G	0.069	87^#^	
NFKBIE	NM_004556	3	G1659C†	0.0144	84^#^	
NFKBIE	NM_004556	3	U1644G†	0.0333	98^#^	
RASSF1	NM_170714	3	A1907U	0.0629	64^#^	
RASSF1	NM_170714	3	G1904A	0.0659	64^#^	
RXRB	NM_021976	3	U2066G	0.0268	59^#^	rs2744537
RXRB	NM_021976	3	U2053A	0.0452	73^#^	rs5030979
PCBP4	NM_001174100	3	U1862G	0.0401	93^#^	
PCBP4	NM_001174100	3	C1790G	0.0413	59^#^	
MTA2	NM_004739	5	A227G	0.0462	73^#^	
BECN1	NM_003766	3	U1970C†	0.012	100^#^	
CTSB	NM_147782	3	A2561G	0.0569	100^$^	
HTT	NM_002111	3	C9948G†	0.0987	100^#^	rs362305
HTT	NM_002111	3	U9947C	0.0225*	100^#^	
BECN1	NM_003766	3	G2053A	0.0329*	98^#^	rs11552193
LMNB2	NM_032737	3	A3713G	0.0554*	59^#^	
LMNB2	NM_032737	3	U3662C	0.0638*	57^#^	
CTSB	NM_147782	3	A2581G	0.0925*	50^$^	
MTA2	NM_004739	5	C267G	0.1035*	53^#^	

^a^ SNVs that were predicted by both d_max_ and r_min_ measures are highlighted with †.

^b^ The p-value corresponding to the r_min_ measure is highlighted with *.

^c^ The conserved RNA secondary structure predicted by CMfinder and RNAz program (through our in-house pipeline [39]) are highlighted with the symbols ^#^ and ^$^, respectively.

Out of the 472 disruptive SNVs obtained from the secondary structure analysis (before intersecting with the cancer-associated genes), 199 overlap with SNPs from dbSNP (build 135). Of these 199 SNVs, 17 are in linkage dis-equilibrium (LD) with other SNPs that are located proximal (+/−200 nts) to the SNV position. These 17 pairs were tested with RNAsnp to check whether the SNP in LD with (disruptive) SNV is a structure-stabilizing haplotype [60]. Of these 17 pairs, five were predicted to cause no significant structural changes, which could be possible structure-stabilizing haplotypes; whereas the other 12 pairs have shown significant structural changes (see File S3.xls).

### Effect of SNVs on microRNA target sites

Screening all human mature miRNAs against all identified SNVs with flanking sequence yields 2×59,810,180 possible combinations. The initial TargetScan step is a conservative filter and reduces the set of SNV-miRNA pairs to 0.2% of this. We then apply miRanda as a second target prediction method, followed by a set of filters. The distribution of *lr* values in the *alter* set is shown in Figure S3, only cases with relative changes larger than the described cut off are considered (see Materials and Methods). Figure 2 shows the different steps with individual counts of putative interaction sites at each stage. This gives us 490 SNVs in 447 genes predicted to affect 707 interactions with 344 miRNAs (see File S4.xlsx). After intersection with known cancer-associated genes (final step in the pipeline, Figure 1), we find 124 SNVs and 148 miRNAs to be involved in 186 interactions that differ with the mutation. These SNVs that induce putative miRTS changes can be further classified into those enhancing interaction with the mutant (80) or wild-type (52) variant.

Table 3 lists all genes that contain more than one miRTS predicted to be changed between wild-type and SNV. This includes examples where the same SNV changes the target site for different mature miRNAs from the same family, but also examples where different SNVs within the gene cause a gain or loss of a miRTS. Similarly, all miRNAs with more than two changed target sites are presented in Table 4. It lists members of the miR-29 family which have previously been reported to act as tumor suppressors as well as oncogenes (see [61] for a review).

**Table 3:** List of genes which have more than one miRNA target site change (*create*, *alter*, *destroy*) in their UTRs.

Gene	mRNA	UTR	SNV	dbSNP 135	miRNA(s)
ACTR3	NM_005721	3	G2078A	rs6642	**miR-662**
ACTR3	NM_005721	5	U232G		*miR-18a-3p*
AMD1	NM_001033059	5	A262G		*miR-1236-3p*
AMD1	NM_001634	5	A263G		*miR-1236-3p*
ARL5B	NM_178815	3	A947U		*miR-409-3p*
ARL5B	NM_178815	3	G2609U	rs12098599	**miR-362-3p**
ARL5B	NM_178815	3	U946C		*miR-124-5p*, *miR-599*
BCL2L13	NM_001270731	3	U1850G	rs725768	*miR-361-3p*
BCL2L13	NM_001270731	3	U2269A	rs74932682	*miR-519b-3p*
BCL7A	NM_001024808	3	G1801C		**miR-650**
BCL7A	NM_001024808	3	U1804C		**miR-650**
CALM1	NM_006888	3	A1872G	rs63576962	*miR-211-5p*
CALM1	NM_006888	3	C2472G		*miR-29a-3p*, *miR-29b-3p*, *miR-29c-3p*
CBX1	NM_001127228	3	A1839U	rs6847	**miR-548b-5p**, **miR-548c-5p**, **miR-548d-5p**
CBX5	NM_012117	3	A2592G		*miR-887*
CBX5	NM_012117	3	C11158U		*miR-654-5p*
CCND2	NM_001759	3	C2086U		*miR-21-3p*
CCND2	NM_001759	3	G5917U		**miR-139-5p**
CKLF	NM_016326	5	U72G		*miR-29a-3p*, *miR-29b-3p*, *miR-29c-3p*
EIF4EBP2	NM_004096	3	C5092G		**miR-15b-5p**, **miR-16-5p**, **miR-195-5p**, **miR-424-5p**, **miR-503-5p**, **miR-646**
IGFBP5	NM_000599	3	G2493C		*miR-675-5p*
IGFBP5	NM_000599	3	G3898U	rs13403592	*miR-29a-3p*, *miR-29b-3p*, *miR-29c-3p*
JAK1	NM_002227	3	U5007A		**miR-106a-5p**, **miR-106b-5p**, **miR-17-5p**, **miR-20a-5p**, **miR-20b-5p**, **miR-519a-3p**, **miR-519b-3p**, **miR-519c-3p**, **miR-519d**, **miR-520g**, **miR-520h**, **miR-526b-3p**, **miR-93-5p**
KLF10	NM_005655	3	C2615A	rs6935	**miR-337-3p**, **miR-614**
KRAS	NM_033360	3	U1049G	rs712	*miR-151a-5p*, **miR-877-5p**
KREMEN1	NM_001039570	3	A5345C		**miR-519a-3p**, **miR-520b**, **miR-520c-3p**, **miR-636**
LMNB2	NM_032737	3	C2928G		**miR-423-5p**
LMNB2	NM_032737	3	C2929A		**miR-423-5p**
NCK2	NM_003581	3	G1974U		**miR-137**, *miR-488-3p*
NDUFB7	NM_004146	5	G39C	rs45628939	*miR-192-5p*, *miR-215*
NR1D2	NM_005126	3	A2782G		*miR-338-5p*
NR1D2	NM_005126	3	U2791C		*miR-504*
P4HA1	NM_001017962	5	C174G		*miR-412*
P4HA1	NM_001142595	5	C174G		*miR-412*
PANX1	NM_015368	3	G2082A	rs1046805	**miR-10a-5p**, **miR-10b-5p**
PBX1	NM_001204961	3	A4035G		*miR-188-5p*
PBX1	NM_001204961	3	G2877U		*miR-187-5p*, *miR-222-3p*
PBX1	NM_001204963	3	A3249G	rs12723035	*miR-326*, *miR-330-5p*
PDPK1	NM_002613	3	U2034G		**miR-504**, *miR-518a-3p*, *miR-518b*, *miR-518d-3p*, *miR-518f-3p*
PRKAB2	NM_005399	3	U4199G		*miR-150-5p*, *miR-532-3p*
PTPN1	NM_002827	3	G2125A	rs118042879	**miR-141-5p**, **miR-942**
RAP1A	NM_002884	3	C1100A	rs6573	**miR-135a-3p**, *miR-196a-5p*, *miR-196b-5p*
SDC4	NM_002999	3	U1874G		*miR-361-3p*
SDC4	NM_002999	3	U1878G		**miR-548d-3p**
SESN2	NM_031459	3	A2864C	rs10494394	**miR-182-5p**, *miR-92a-1-5p*, **miR-96-5p**
SLC39A6	NM_012319	3	C3543U		**miR-144-3p**
SLC39A6	NM_012319		G3545U		**miR-101-3p**, **miR-139-5p**, **miR-144-3p**
SMAD5	NM_005903	3	C2825A		**miR-124-3p**, *miR-500a-3p*, *miR-501-3p*, *miR-502-3p*, **miR-506-3p**
SMNDC1	NM_005871	3	G1228A	rs1050755	**miR-329**, **miR-362-3p**
SUZ12	NM_015355	3	C2473G		**miR-30a-3p**, **miR-30d-3p**, **miR-30e-3p**, *miR-452-5p*
SUZ12	NM_015355	3	G2475U		**miR-30a-3p**, **miR-30d-3p**, **miR-30e-3p**, *miR-595*
SUZ12	NM_015355	3	U2474A		**miR-30a-3p**, **miR-30e-3p**
TNFRSF19	NM_001204458	3	A2148U		*miR-766-3p*
TNFRSF19	NM_001204458	3	A2452U	rs79570196	*miR-26a-5p*
TOMM20	NM_014765	3	A3198C		*miR-149-3p*
TOMM20	NM_014765	3	U3378G		**miR-129-1-3p**, **miR-129-2-3p**
TOMM20	NM_014765	3	U3379G		*miR-150-5p*, *miR-532-3p*

The miRNA IDs are boldface if the interaction is predominant in the wild-type (*destroy* or *alter* with 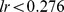) and italics if the interaction is specific to the mutant (*create* or *alter* with 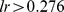); the hsa- prefix is omitted for brevity.

**Table 4:** List of miRNAs with more than two targets in the filtered data set.

Gene	mRNA	UTR	SNV	miRNA	ΔG_WT_	ΔG_SNV_	dbSNP 135
CALM1	NM_006888	3	C2472G	hsa-miR-29a-3p	N/A	−16.70	
CKLF	NM_016326	5	U72G	hsa-miR-29a-3p	N/A	−20.70	
IGFBP5	NM_000599	3	G3898U	hsa-miR-29a-3p	N/A	−13.83	rs13403592
CALM1	NM_006888	3	C2472G	hsa-miR-29b-3p	N/A	−18.12	
CKLF	NM_016326	5	U72G	hsa-miR-29b-3p	N/A	−18.76	
IGFBP5	NM_000599	3	G3898U	hsa-miR-29b-3p	N/A	−11.93	rs13403592
CALM1	NM_006888	3	C2472G	hsa-miR-29c-3p	N/A	−15.13	
CKLF	NM_016326	5	U72G	hsa-miR-29c-3p	N/A	−18.83	
IGFBP5	NM_000599	3	G3898U	hsa-miR-29c-3p	N/A	−12.71	rs13403592
SUZ12	NM_015355	3	C2473G	hsa-miR-30a-3p	−12.66	−8.97	
SUZ12	NM_015355	3	G2475U	hsa-miR-30a-3p	−12.66	−6.32	
SUZ12	NM_015355	3	U2474A	hsa-miR-30a-3p	−12.66	−6.86	
SUZ12	NM_015355	3	C2473G	hsa-miR-30e-3p	−12.53	−8.02	
SUZ12	NM_015355	3	G2475U	hsa-miR-30e-3p	−12.53	−6.19	
SUZ12	NM_015355	3	U2474A	hsa-miR-30e-3p	−12.53	−6.73	
BCL2L13	NM_001270731	3	U1850G	hsa-miR-361-3p	N/A	−17.85	rs725768
SDC4	NM_002999	3	U1874G	hsa-miR-361-3p	−15.88	−20.18	
SOX4	NM_003107	3	G4753A	hsa-miR-361-3p	−20.76	−14.48	rs11556729
BCL2L13	NM_001270731	3	U2269A	hsa-miR-519b-3p	N/A	−11.07	rs74932682
JAK1	NM_002227	3	U5007A	hsa-miR-519b-3p	−16.69	−12.31	
OSMR	NM_003999	3	C4534U	hsa-miR-519b-3p	N/A	−13.92	
FAM46C	NM_017709	3	A1459G	hsa-miR-614	−17.89	−22.28	rs2066411
KLF10	NM_005655	3	C2615A	hsa-miR-614	−22.30	−17.54	rs6935
RHEB	NM_005614	3	A1229G	hsa-miR-614	N/A	−15.19	

Of the 148 miRNAs (responsible for 186 putative interactions) in our final candidate set, 89 are *lung cancer-associated miRNAs* (in 117 interactions) (indicated in File S4.xlsx). Table 5 lists all 14 putative target sites in our final candidate set that include *NSCLC-associated miRNAs*. Notably, the list includes four miRNAs with more than one predicted target changed. For miR-184 one target site is created while another one is weakened upon introduction of the mutation. Moreover, miR-30a, d, and e are predicted to target the 3′ UTR of *SUZ12* gene. However, the predicted interactions are likely to be functional in the wild-type and lost in the mutant due to SNV-induced changes at the seed region. SUZ12 has previously been shown to be directly targeted by miR-200b and inhibition of this miRNA increases the formation of cancer stem cells (CSCs) [62], which contribute to tumor aggressiveness. The loss of miR-30 regulation by (one of) the three adjacent SNVs in the seed of the target site could have a similar effect in NSCLC.

**Table 5:** List of target predictions of *NCSLC-associated miRNAs* derived from the microRNA body map [45].

Gene	mRNA	UTR	SNV	miRNA	ΔG_WT_	ΔG_SNV_	dbSNP 135
DHCR24	NM_014762	3	A4192C	hsa-miR-7-5p	N/A	−11.85	
EIF4EBP2	NM_004096	3	C5092G	hsa-miR-15b-5p	−16.10	−10.65	
EIF4EBP2	NM_004096	3	C5092G	hsa-miR-16-5p	−18.20	−13.97	
EIF4EBP2	NM_004096	3	C5092G	hsa-miR-195-5p	−17.63	−12.23	
KIF3B	NM_004798	3	G5433A	hsa-miR-184	−21.40	−14.40	rs41289846
MED16	NM_005481	5	A129U	hsa-miR-184	N/A	−20.65	
SUZ12	NM_015355	3	C2473G	hsa-miR-30a-3p	−12.66	−8.97	
SUZ12	NM_015355	3	C2473G	hsa-miR-30d-3p	−11.68	−8.65	
SUZ12	NM_015355	3	C2473G	hsa-miR-30e-3p	−12.53	−8.02	
SUZ12	NM_015355	3	G2475U	hsa-miR-30a-3p	−12.66	−6.32	
SUZ12	NM_015355	3	G2475U	hsa-miR-30d-3p	−11.68	−5.57	
SUZ12	NM_015355	3	G2475U	hsa-miR-30e-3p	−12.53	−6.19	
SUZ12	NM_015355	3	U2474A	hsa-miR-30a-3p	−12.66	−6.86	
SUZ12	NM_015355	3	U2474A	hsa-miR-30e-3p	−12.53	−6.73	

Furthermore, for 48 SNVs the predicted miRTSs were found to be located inside the local region where a significant secondary structural change was predicted by RNAsnp. Of these, 15 SNVs were located in the cancer-associated genes (see Table 6). Based on the previous studies [63], [64], we speculate that the SNV-induced miRTS change along with the secondary structural changes in and around the miRTS can potentially affect the binding of predicted miRNA.

**Table 6:** List of predicted miRNA target site changes that overlap with RNAsnp predictions.

Gene	mRNA	UTR	SNV	miRNA	ΔG_WT_	ΔG_SNV_	RNAsnp (p-value)^a^
DHCR24	NM_014762	3	A4192C	hsa-miR-7-5p	N/A	−11.85	0.0159
EIF4EBP2	NM_004096	3	C5092G	hsa-miR-15b-5p	−16.10	−10.65	0.0341
EIF4EBP2	NM_004096	3	C5092G	hsa-miR-16-5p	−18.20	−13.97	0.0341
EIF4EBP2	NM_004096	3	C5092G	hsa-miR-195-5p	−17.63	−12.23	0.0341
EIF4EBP2	NM_004096	3	C5092G	hsa-miR-424-5p	−16.13	−12.18	0.0341
EIF4EBP2	NM_004096	3	C5092G	hsa-miR-503-5p	−17.19	−12.42	0.0341
EIF4EBP2	NM_004096	3	C5092G	hsa-miR-646	−13.75	−9.56	0.0341
ATP6V1C2	NM_144583	3	G2321C	hsa-miR-615-3p	N/A	−18.91	0.0483
NOP10	NM_018648	3	G432A	hsa-miR-342-3p	−22.81	−18.51	0.0518
RAD21	NM_006265	3	G3118U	hsa-miR-361-5p	−11.01	N/A	0.0696
PANX1	NM_015368	3	G2082A	hsa-miR-10a-5p	−14.54	−7.67	0.0704
PANX1	NM_015368	3	G2082A	hsa-miR-10b-5p	−13.85	N/A	0.0704
CCND2	NM_001759	3	G5917U	hsa-miR-139-5p	−13.21	−7.52	0.0726
SESN2	NM_031459	3	A2864C	hsa-miR-92a-1-5p	−14.56	−21.55	0.0818
SESN2	NM_031459	3	A2864C	hsa-miR-96-5p	−14.76	−9.52	0.0818
SESN2	NM_031459	3	A2864C	hsa-miR-182-5p	−19.86	−15.36	0.0818
PPA1	NM_021129	5	G92U	hsa-miR-378a-5p	−15.90	−20.85	0.083
SLC39A6	NM_012319	3	G3545U	hsa-miR-144-3p	−11.40	−8.09	0.0832
SLC39A6	NM_012319	3	G3545U	hsa-miR-101-3p	−20.37	−14.03	0.0832
SLC39A6	NM_012319	3	G3545U	hsa-miR-139-5p	−20.37	−14.03	0.0832
PPA2	NM_006903	3	A983U	hsa-miR-139-3p	N/A	−18.24	0.0841
TNFRSF19	NM_001204458	3	A2148U	hsa-miR-766-3p	N/A	−14.71	0.0874
SLC39A6	NM_012319	3	C3543U	hsa-miR-144-3p	−11.40	−6.70	0.0924
CRYL1	NM_015974	3	U1350A	hsa-miR-330-5p	N/A	−19.74	0.0487*
HIPK2	NM_001113239	3	U7743G	hsa-miR-181a-2-3p	N/A	−14.08	0.0581*

^a^ SNV predicted by r_min_ measure is highlighted with *.

### Functional analysis of genes predicted with SNVs' effect on UTRs

To illustrate how the candidate SNVs obtained from our pipeline can be further analyzed for potential functionality and co-operability, we investigated the resulting sets from miRNA and RNAsnp analyses individually as well as their combination, each before and after intersection with cancer-related genes. More precisely, the following six gene sets have been tested by Ingenuity Pathways Analysis (see Table 7): 490 SNVs corresponding to 447 genes from miRNA analysis (miRNA in all genes), 124 SNVs corresponding to 104 genes that overlap with our cancer gene set (miRNA in cancer-related genes), 472 SNVs associated with 408 genes from RNAsnp analysis (RNAsnp in all genes), 111 SNVs corresponding to 89 genes that intersect with our cancer gene set (RNAsnp in cancer-related genes), a unique gene list obtained after combination of 447 genes from miRNA analysis and 408 genes from RNAsnp analysis (miRNA and RNAsnp overlap in all genes), and a unique cancer gene list obtained from 104 genes from miRNA and 89 genes form RNAsnp analysis respectively (miRNA and RNAsnp overlap in cancer-related genes).

**Table 7:** Summary of pathway analysis results using Ingenuity pathway analysis software.

miRNA in all genes	miRNA in cancer related genes	RNAsnp in all genes	RNAsnp in cancer related genes	miRNA and RNAsnp overlap in all genes	miRNA and RNAsnp overlap in cancer related genes
**Top 3 networks**					
1. Cell Death and Survival, Cardiovascular System Development and Function, Organismal Development (44)	1. Cell Death and Survival, Cellular Growth and Proliferation, DNA Replication, Recombination, and Repair (34)	1. Skeletal and Muscular System Development and Function, Cell Death and Survival, Cardiovascular System Development and Function (40)	1. Cellular Growth and Proliferation, Cell Death and Survival, Cellular Development (42)	1. Cell Signaling, Nucleic Acid Metabolism, Small Molecule Biochemistry (38)	1. Gene Expression, Cell Death and Survival, Cancer (43)
2. Cell Death and Survival, Cell-To-Cell Signaling and Interaction, Nervous System Development and Function (29)	2. Cellular Growth and Proliferation, Cell Death and Survival, Cardiovascular System Development and Function (16)	2. Cell Death and Survival, Cellular Function and maintenance, Cell Morphology (40)	2. Cell Death and Survival, Cellular Assembly and Organization, Cell Cycle (37)	2. Cellular Growth and Proliferation, Cell Morphology, Cellular Assembly and Organization (34)	2. Cellular Growth and Proliferation, Cell Death and Survival, Cellular Assembly and Organization (41)
3. Hematological Disease, Immunological Disease, Cellular Development (27)	3. Cardiovascular Disease, Gene Expression, Organismal Development (14)	3. Cellular Assembly and Organization, Post-Translational Modification, Cellular Movement (32)	3. Gene Expression, Cellular Growth and Proliferation, Embryonic Development (24)	3. Cellular Movement, Cell Death and Survival, Cardiovascular System Development and Function (34)	3. Cell Death and Survival, Dermatological Diseases and Conditions, Cellular Development (34)
**Top 3 diseases and disorders**					
1. Infectious Disease (1.75E-4–4.85E-2)	1. Cancer (4.36E-6–4.90E-2)	1. Cancer (2.03E-4–4.41E-2)	1. Cancer (9.42E-8–1.27E-2)	1. Infectious Disease (1.04E-5–4.31E-2)	1. Cancer (8.23E-10–1.22E-2)
2. Cancer (1.42E-3–4.71E-2)	2. Hematological Disease (1.16E-4–4.05E-2)	2. Endocrine System Disorders (4.95E-4–2.38E-2)	2. Hematological Disease (1.46E-5–1.27E-2)	2. Cancer (1.93E-4–4.31E-2)	2. Hematological Disease (1.48E-8–1.22E-2)
3. Hepatic System Disease (1.42E-3–2.38E-2)	3. Endocrine System Disorders (1.36E-4–3.34E-2)	3. Reproductive System Disease (4.95E-4–4.08E-2)	3. Gastrointestinal Disease (1.08E-4–1.27E-2)	3. Hepatic System Disease (3.37E-4–4.31E-2)	3. Infectious Disease (4.22E-6–8.87E-3)
**Top 3 molecular and cellular functions**					
1. Protein Synthesis (4.99E-6–2.09E-3)	1. Cellular Growth and Proliferation (4.03E-9–4.65E-2)	1. Cellular Growth and Proliferation (6.76E-6–4.41E-2)	1. Cellular Growth and Proliferation (1.18E-17–1.27E-2)	1. Cellular Growth and Proliferation (3.14E-7–4.31E-2)	1. Cellular Growth and Proliferation (5.68E-25–1.22E-2)
2. RNA Post-Transcriptional Modification (5.32E-4–2.38E-2)	2. Cell Death and Survival (7.07E-8–4.68E-2)	2. Cell Death and Survival (7.24E-6–4.41E-2)	2. Cell Death and Survival (4.08E-17–1.27E-2)	2. Cell Death and Survival (6.1E-7–4.31E-2)	2. Cell Death and Survival (1.09E-19–1.22E-2)
3. RNA Damage and Repair (5.67E-4–5.67E-4)	3. Cellular Development (1.02E-6–4.00E-2)	3. Cellular Assembly and Organization (4.83E-5–4.41E-2)	3. Cellular Development (5.79E-14–1.27E-2)	3. Protein Synthesis (2.39E-5–3.73E-2)	3. Cellular Development (1.89E-18–1.22E-2)
**Top 3 canonical pathways**					
1. EIF2 Signaling (1.61E-5)	1. Molecular Mechanisms of Cancer (4.14E-7)	1. LPS-stimulated MAPK Signaling (3.37E-5)	1. LPS-stimulated MAPK Signaling (8.4E-10)	1. iNOS Signaling (4.32E-4)	1. Molecular Mechanisms of Cancer (3.11E-12)
2. mTOR Signaling (4.31E-4)	2. Insulin Receptor Signaling (2.47E-6)	2. iNOS Signaling (3.56E-4)	2. Molecular Mechanisms of Cancer (2.65E-8)	2. EIF2 Signaling (4.52E-4)	2. Glucocorticoid Receptor Signaling (2.88E-9)
3. Insulin Receptor Signaling (9.85E-4)	3. IGF-1 Signaling (4.17E-6)	3. Germ Cell-Sertoli Cell Junction Signaling (6.75E-4)	3. IL-6 Signaling (5.27E-8)	3. mTOR Signaling (8.85E-4)	3. LPS-stimulated MAPK Signaling (1.9E-8)

The numbers at the end of each cell represent the p-values, but for the top networks it is the p-score (−log_10_ *p-value*).

**Figure 4:**
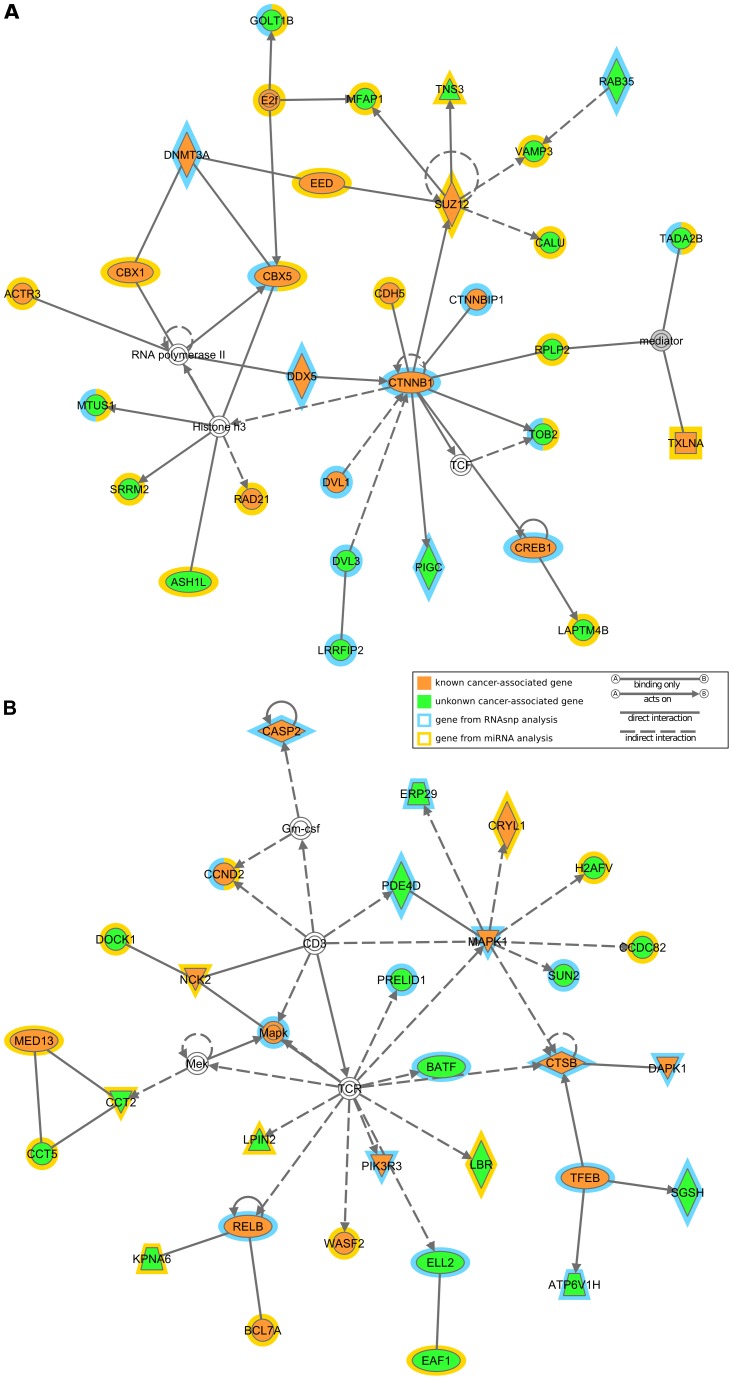
Network Analysis of genes predicted to have SNVs' effect on UTRs. The networks represent the interaction between genes that were predicted to have SNVs' effect on UTRs from miRNA and RNAsnp analysis (see Table 7, column 5). The gene nodes were colored to differentiate the known (orange) and unknown (green) cancer-associated genes, and the color outside the node indicates whether the gene comes from miRNA (yellow) or RNAsnp (blue) or both.

Based on significant p-values obtained from each of our analyses, we have listed the top 3 networks, diseases and disorders, molecular and cellular functions, and canonical pathways in Table 7. Our results indicate that the top networks identified from our six gene set analyses are highly enriched with *cell death and survival* as well as *cellular growth and proliferation* (see Table 7). Figure 4 shows the network of those two cases which were predicted using the genes from the combination of miRNA and RNAsnp analyses, whereas the networks from other gene sets are shown in Figure S4. The networks shown in Figure 4 contain several genes from Table 2 and 3 which were predicted to have more than one disruptive SNV, and also genes from Table 6 for which predictions from miRNA and RNAsnp analyses overlap. We predict that these genes have a higher chance of being disrupted by the SNVs in UTRs, which might cause a change in protein translation and thereby disrupt the interaction of this protein with others.

The top diseases and disorders associated with the gene sets predominantly include cancer. In addition, top three canonical pathways related to the gene sets are molecular mechanisms of cancer, LPS-stimulated MAPK signaling, IL-6 signaling, iNOS signaling, EIF2 signaling and mTOR signaling.

It should be noted that the enrichment for cancer and related molecular functions is found in our miRNA and RNAsnp gene sets even before intersecting with the list of cancer-associated genes (see Table 7).

## Discussion

With the help of whole-genome sequencing technology, the complete genome of a cancer cell can be sequenced effectively to identify somatic single nucleotide variants (SNVs) [65]. To date, more than 50 different cancer types and/or sub types have been sequenced [2]. The lung cancer genome was first sequenced in 2010 [66], which reports that the somatic variants were present in both coding and non-coding (UTR and other non-coding RNAs) transcribed regions, which constitute 0.6% and 0.8% respectively of the total somatic mutations identified (22,910). In a recent study, transcriptome-wide sequencing of non-small cell lung cancer (NSCLC) type with wild-type and mutant *KRAS* revealed 73,717 SNVs that consisted of both germ-line and somatic variants. Of these SNVs, 29,290 were located in the UTRs of mRNAs that correspond to 6462 genes.

We have developed a comprehensive computational pipeline to predict the effects of SNVs located in the UTRs that can potentially affect the post-transcriptional regulation, through SNV-induced secondary structure changes in the UTRs or changes in miRTSs within UTRs. Using this pipeline, we predicted 472 out of 29,290 UTR SNVs to have significant effect on the local RNA secondary structure of UTRs (corresponding to 408 genes). Additionally, 490 out of 29,290 UTR SNVs were predicted to cause changes in a miRNA target site within the UTRs of 447 genes. Of these 490 SNVs, 124 were present in 104 genes that were previous known to be cancer-associated. For these 104 genes, 148 miRNAs were predicted to bind either in the wild-type or mutant. We found 89 out of these 148 miRNAs overlap with *lung cancer-associated miRNAs*, while eight miRNAs are associated specifically to NSCLC.

Taken together, all these disruptive SNVs, which were predicted to affect secondary structure or miRNA target sites, were present in 803 different genes; out of which 188 (23.4%) were previously known to be cancer-associated. Notably, this ratio is significantly higher (p-value 0.032, one-sided Fisher's exact test) than the ratio (20.8%) of known cancer-associated genes (n = 1347) in our initial data set of 6462 genes. Similar enrichment (p-value 0.040, one-sided Fisher's exact test) was observed when comparing the ratio of disruptive SNVs in cancer-associated genes compared to all other genes versus the ratio of the data set SNVs in cancer-associated genes compared to all other genes. However, while comparing the ratios separately on the results obtained from RNA secondary structure and miRTS analysis, we did not find any significant difference (data not shown).

Further, the IPA networks analysis (that addresses the biological relationships between genes/gene products) shows that the physical interaction of genes predicted with SNV effect might be involved in *cell death and survival* as well as *cellular growth and proliferation*. However, further analysis of these networks with respect to the topology (e.g., edge counts, neighborhood connectivity, in and out degree) is required. The functional analysis using IPA shows that the genes from our pipeline were involved in canonical pathways such as molecular mechanisms of cancer, IL-6 signaling, LPS-stimulated MAPK signaling pathways, iNOS Signaling, EIF2 signaling and mTOR signaling pathways.

Given the large data set of 29,290 SNVs and the generally high false positive rate of established miRNA target prediction methods, we chose stringent filters in the miRNA analysis. The requirement of a TargetScan seed change, used to reduce the initial set of pairs, is present in 60% of our benchmark data (Table S1). The intersection of candidates in the *alter* and *destroy* sets with Ago CLIP-Seq data is another conservative filter. Due to incompleteness of the data, this filters out some true interactions (as can be seen from Table S1), but gives higher confidence in the remaining candidates (i.e., not all known miRNA interactions actually overlap with the Ago CLIP-Seq peak clusters; but if there is a cluster with BC≥2 there likely is a real interaction). Interactions from the *create* set are not issued to that filter, so the resulting candidates might be biased towards that. Individual filters can be left out or chosen to be more or less conservative depending on the data set at hand. Also, it should be noted that this data set contains both germ-line and somatic variants. In the previous study, no normal cells were sequenced in parallel to the lung adenocarcinomas to remove germ-line variants. Based on the overlap of these SNVs with the dbSNP (build 135) we could estimate that 40% of the 29,290 SNVs are germ-line variants. However, we did not remove those germ-line variants in this analysis because these germ-line variants are also important and may have a role for differences in cancer predisposition and drug response between individuals.

In summary, we hypothesize that the SNVs predicted to cause significant changes in the secondary structure of UTRs or miRNA target sites within UTRs can have the potential to alter the expression of genes linked to cancer-associated pathways, and thereby contribute to the development of cancer. Although we do not provide experimental validation to support these predictions, we have highlighted the significant causative SNVs, which will be helpful for further detailed investigation. As for example, the SNV U1552G that affects the structure of the *cis*-acting regulatory element, selenocysteine insertion sequence (SECIS) (Figure 4), which is associated with the translational control of *GPX3* mRNA. The computational pipeline presented here can be adopted for UTR SNV data from other cancer genome and transcriptome studies.

It is worth considering that the SNVs outside the protein-coding regions can have functional impacts causing altered expression of a gene. This may help identification of new cancer driver mutations.

Future directions include protein binding site predictions on both structured and unstructured parts of the UTRs. Merging with the growing amount of experimental data concerning RNA binding proteins, e.g., CLIP-seq, more general types of data than those related to miRNA targets should provide complementary information. For example, additional ranking of predicted binding site structure disruption. Further, if such data is extracted from disease tissue it should provide yet another complementary layer of data pointing to specific candidates.

## Supporting Information

Figure S1 **Overview of RNAsnp predictions.**
(PDF)

Figure S2 **Distribution of GC-content in regions around (disruptive) SNVs.**
(PDF)

Figure S3 **Histogram of** ***lr*** **values in miRNA analysis** ***alter*** **set.**
(PDF)

Figure S4 **Top three IPA networks for the six different gene sets as described in** **Table 7** **.**
(PDF)

File S1 **All candidates from RNAsnp analysis.** Excel table listing 472 SNVs in 408 genes.
(XLSX)

File S2 **Medium-confidence candidates from RNAsnp analysis.** Subset of File S1, lists 83 SNVs in cancer-associated genes with either d_max_ or r_min_ p-value<0.1 (but not both <0.05).
(XLSX)

File S3 **RNAsnp predicted effect of SNPs in LD with disruptive SNVs.**
(XLSX)

File S4 **Candidates from miRNA analysis.** Excel table listing 490 SNVs in 447 genes predicted to affect target sites of 344 miRNAs, with indication of cancer-association genes and *lung cancer-associated miRNAs*.
(XLSX)

Table S1 **Filtration steps in the miRNA pipeline tested on known examples.**
(PDF)
