# Supplementary material for: Transcriptome-Wide Analysis of UTRs in Non-Small Cell Lung Cancer Reveals Cancer-Related Genes with SNV-Induced Changes on RNA Secondary Structure and miRNA Target Sites
Source: PLoS One. 2014 Jan 8;9(1):e82699. doi: 10.1371/journal.pone.0082699 (PMC3885406; doi:10.1371/journal.pone.0082699)
Supplement: Figure S1 — Overview of RNAsnp predictions. (PDF) [file pone.0082699.s001.pdf]

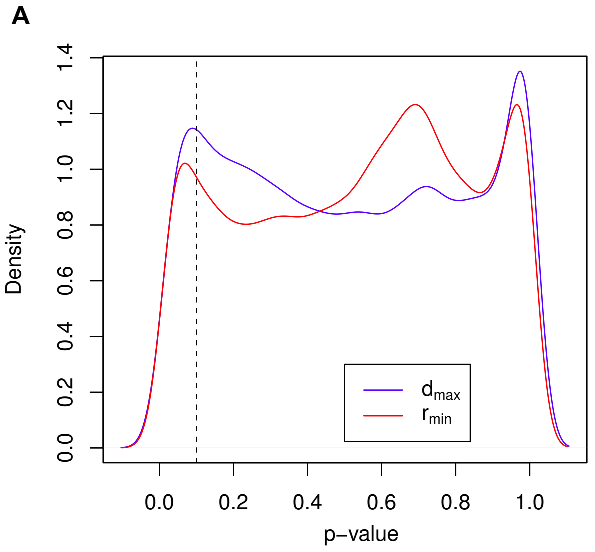

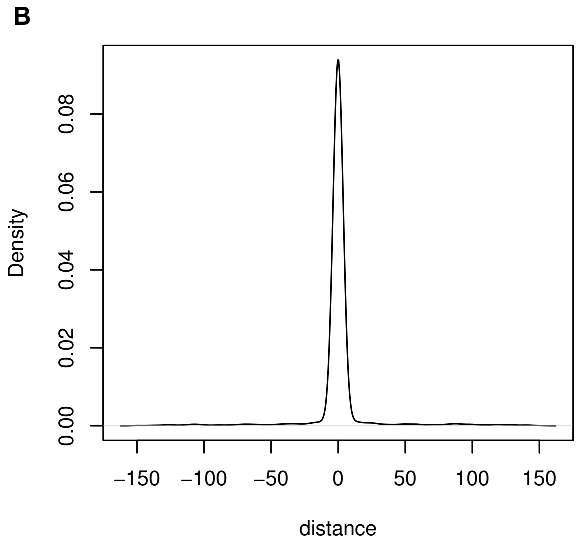

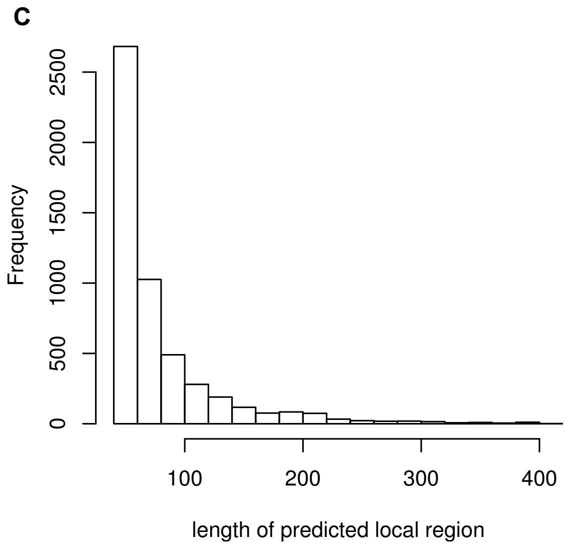

Figure S1: Overview of the RNAsnp results predicted for 29,290 SNVs. a) Distribution of the p-values calculated for 29,290 using d_{max} and r_{min} measures of RNAsnp. The dotted line indicates the threshold value of 0.1. b) Distribution of the distance value between the SNV position and the predicted local region c) Length distribution of the predicted local region.
