# Supplementary material for: Transcriptome-Wide Analysis of UTRs in Non-Small Cell Lung Cancer Reveals Cancer-Related Genes with SNV-Induced Changes on RNA Secondary Structure and miRNA Target Sites
Source: PLoS One. 2014 Jan 8;9(1):e82699. doi: 10.1371/journal.pone.0082699 (PMC3885406; doi:10.1371/journal.pone.0082699)
Supplement: Figure S2 — Distribution of GC-content in regions around (disruptive) SNVs. (PDF) [file pone.0082699.s002.pdf]

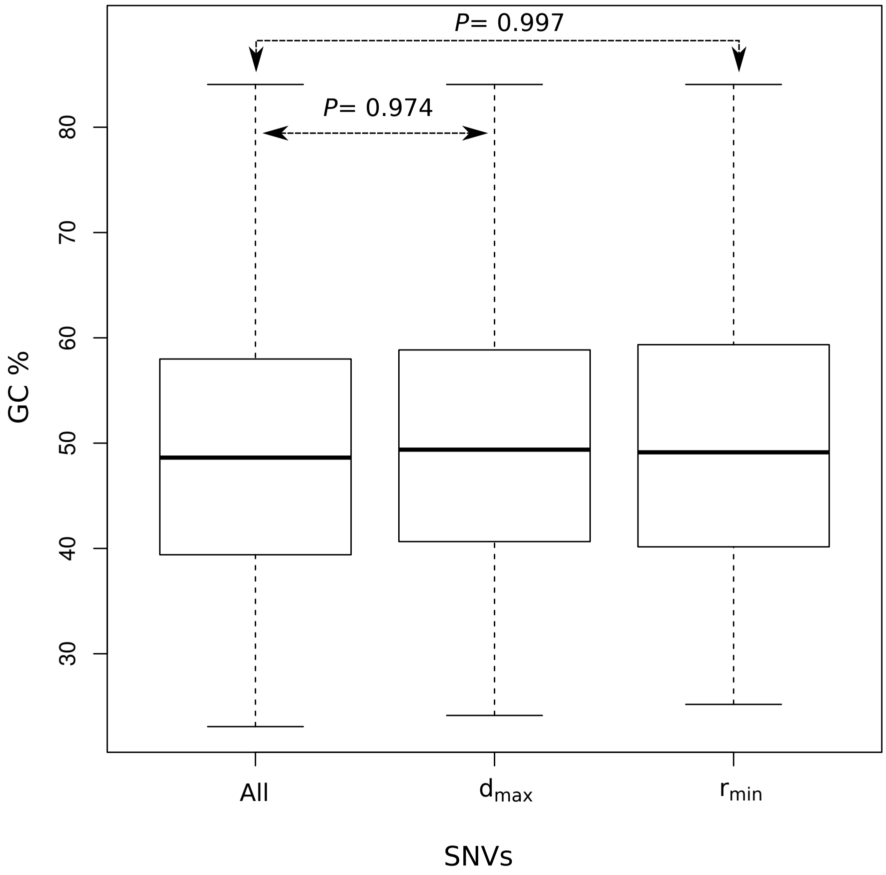

Figure S2: Distribution of GC% computed for all data set SNVs (29,290) and the SNVs predicted to have significant structural changes by d_{max} (3204) and r_{min} (1813) measures of RNAsnp. The plot shows that data set SNVs as well as the disruptive SNVs (from d_{max} and r_{min} measures) are highly enriched in the GC% region ranging from 40 to 60 and there was no significant difference between the GC% distribution of the disruptive SNVs and the data set SNVs (as indicated by the P-values from Kolmogorov–Smirnov test).
