# Supplementary material for: Transcriptome-Wide Analysis of UTRs in Non-Small Cell Lung Cancer Reveals Cancer-Related Genes with SNV-Induced Changes on RNA Secondary Structure and miRNA Target Sites
Source: PLoS One. 2014 Jan 8;9(1):e82699. doi: 10.1371/journal.pone.0082699 (PMC3885406; doi:10.1371/journal.pone.0082699)
Supplement: Figure S3 — Histogram of lr values in miRNA analysis alter set. (PDF) [file pone.0082699.s003.pdf]

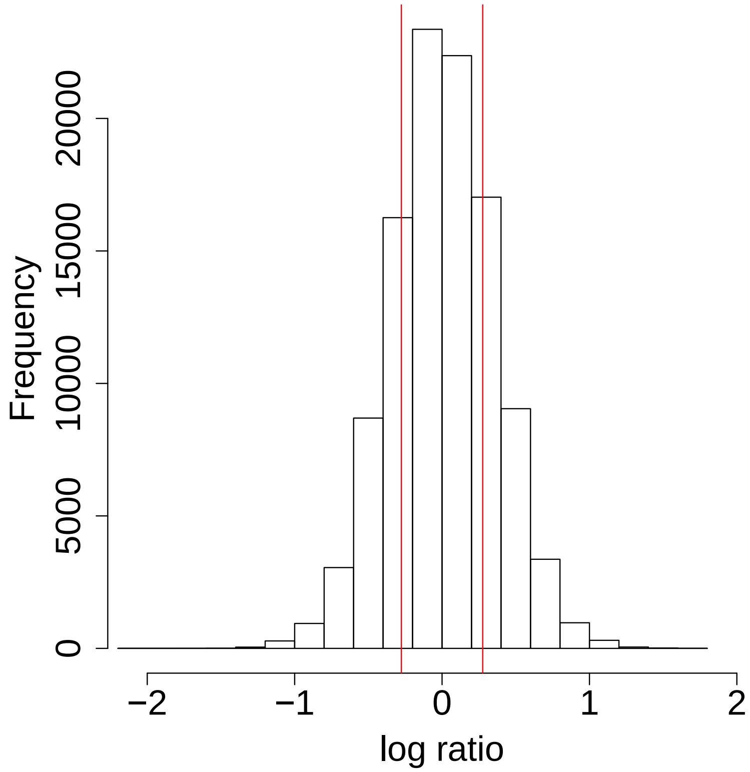

Figure S3: Histogram of the log ratio of WT and SNV variant in *alter* set before intersection with the CLIP-Seq data. Negative values indicate that the wild type has the stronger interaction (lower energy); positive values indicate a stronger binding in the SNV variant. Values around zero refer to interactions with only a slight change in binding and are filtered out. The cut-off value of 0.276 (indicated with red lines) used in this analysis is the mean of absolute log ratio values.
