# Supplementary material for: Transcriptome-Wide Analysis of UTRs in Non-Small Cell Lung Cancer Reveals Cancer-Related Genes with SNV-Induced Changes on RNA Secondary Structure and miRNA Target Sites
Source: PLoS One. 2014 Jan 8;9(1):e82699. doi: 10.1371/journal.pone.0082699 (PMC3885406; doi:10.1371/journal.pone.0082699)
Supplement: Figure S4 — Top three IPA networks for the six different gene sets as described in Table 7 . (PDF) [file pone.0082699.s004.pdf]

i) miRNA in all genes

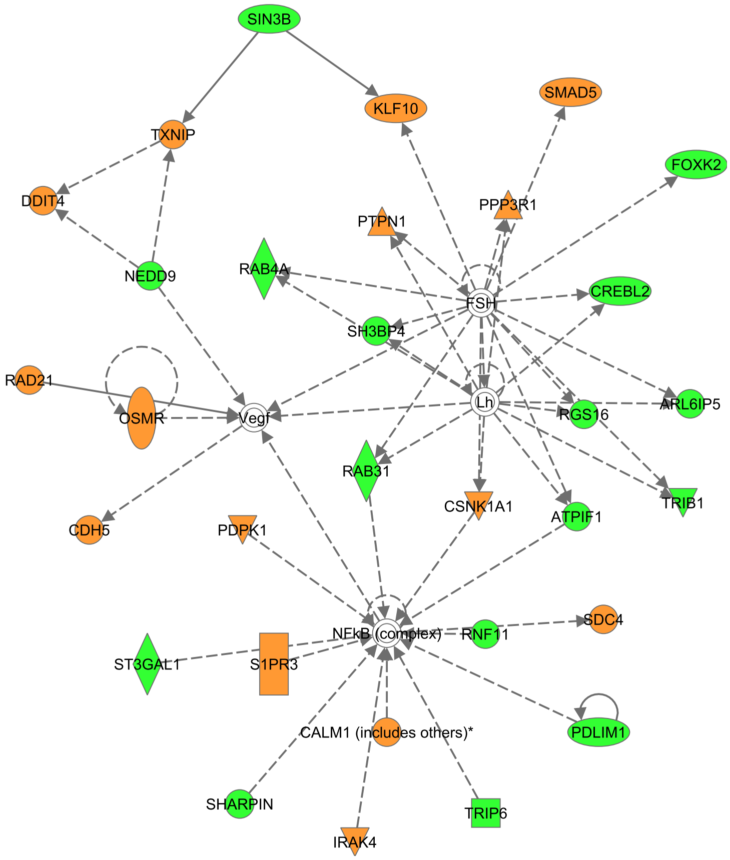

(a)

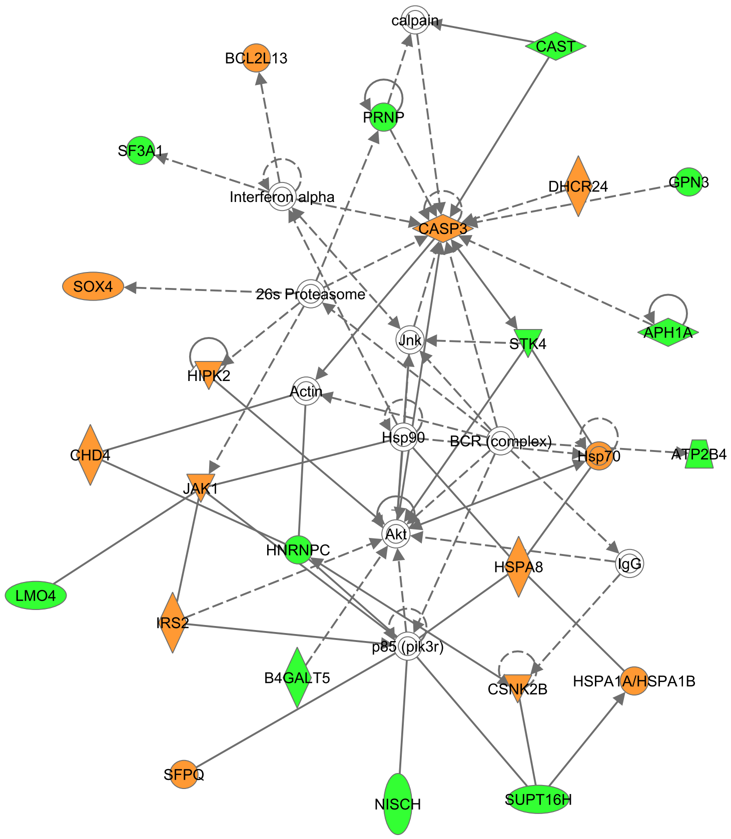

(b)

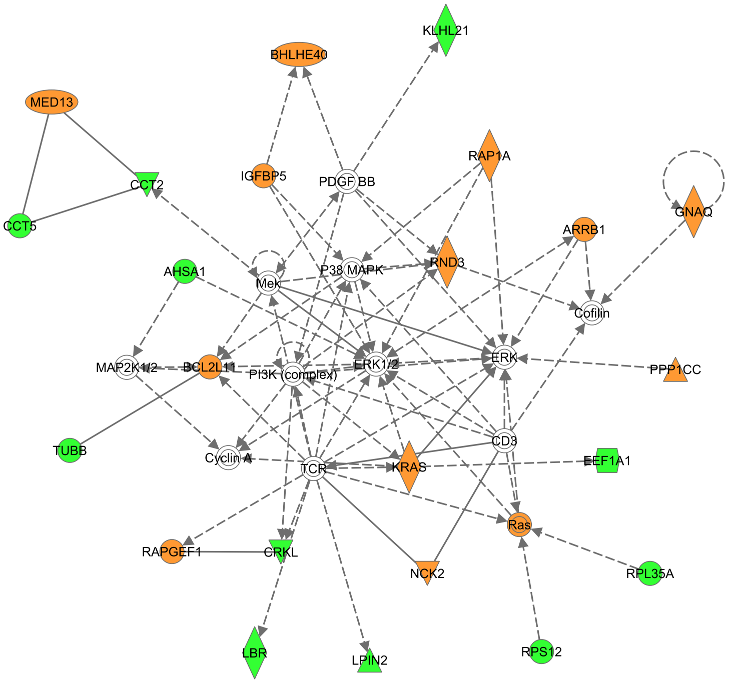

(c)

ii) miRNA in cancer related genes

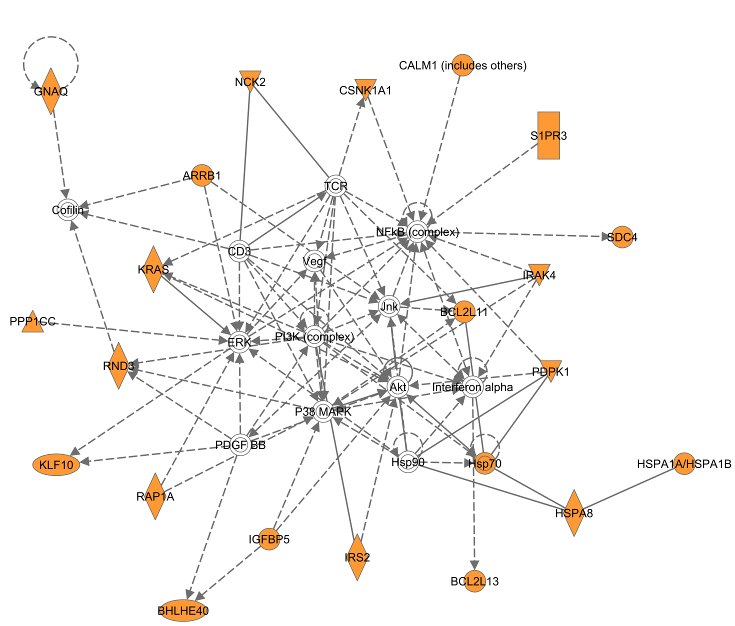

(d)

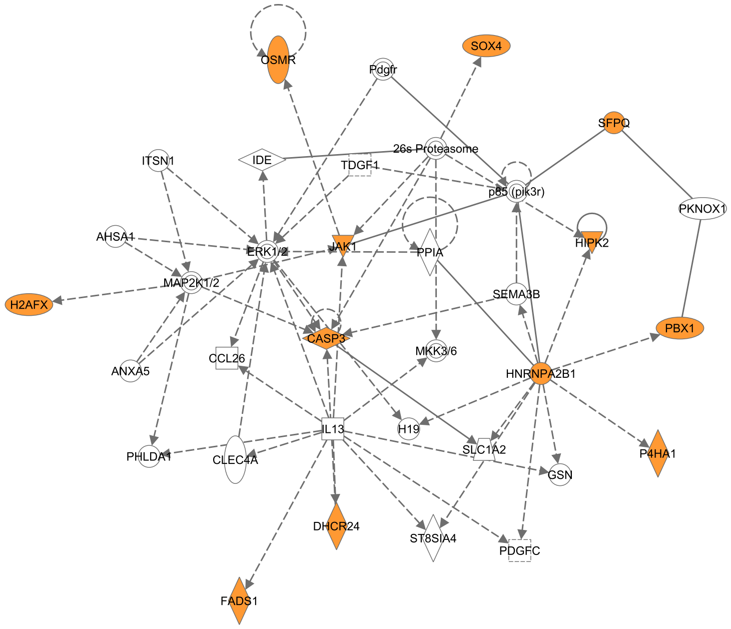

(e)

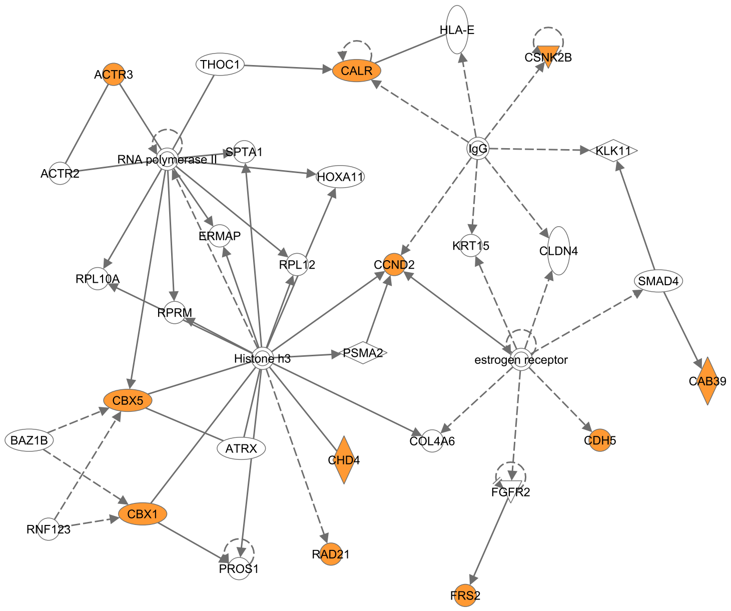

(f)

iii) RNAsnp in all genes

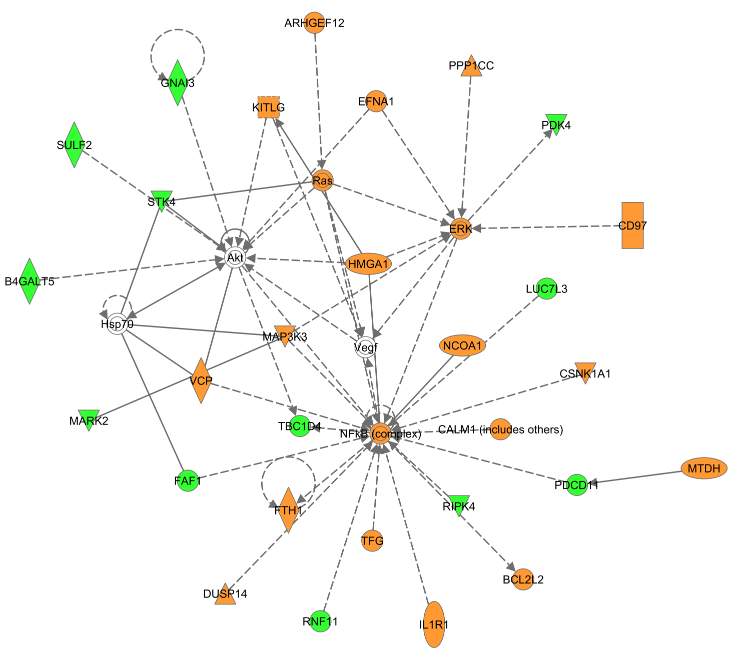

(g)

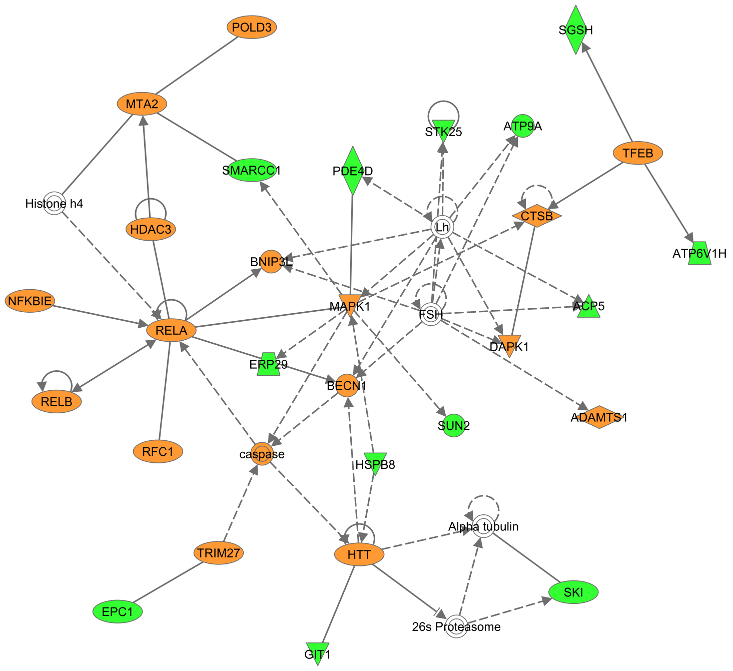

(h)

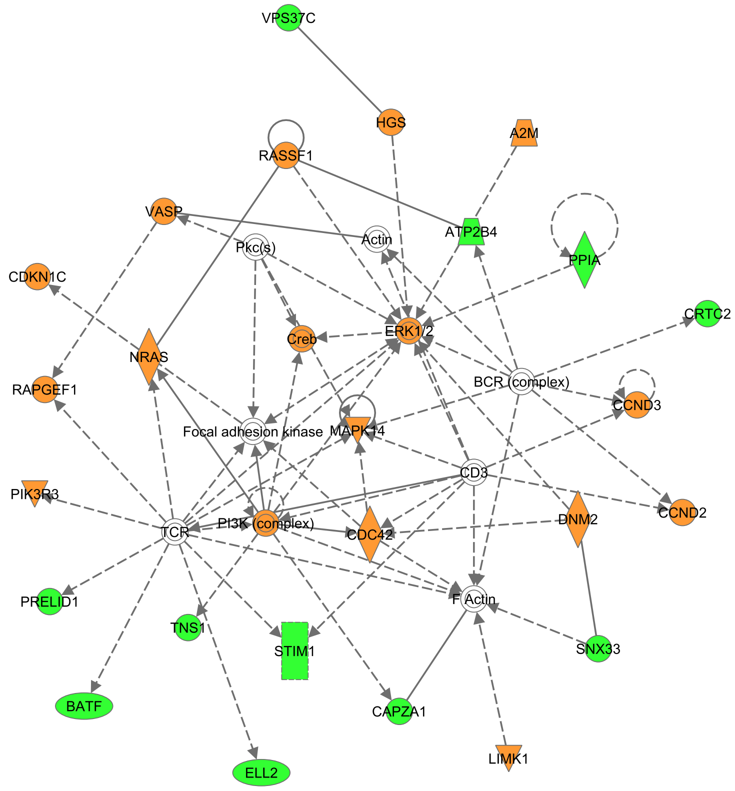

(i)

iv) RNAsnp in cancer related genes

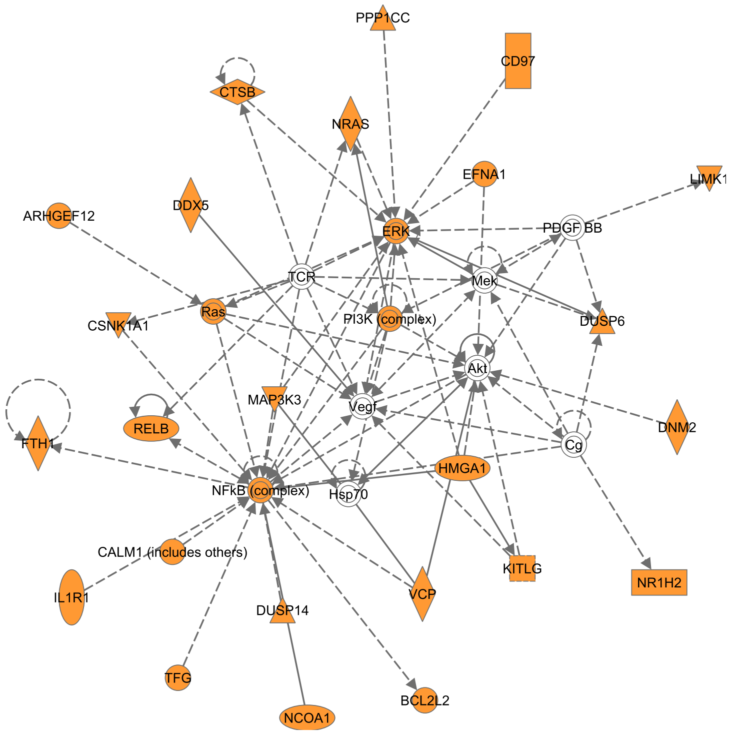

(j)

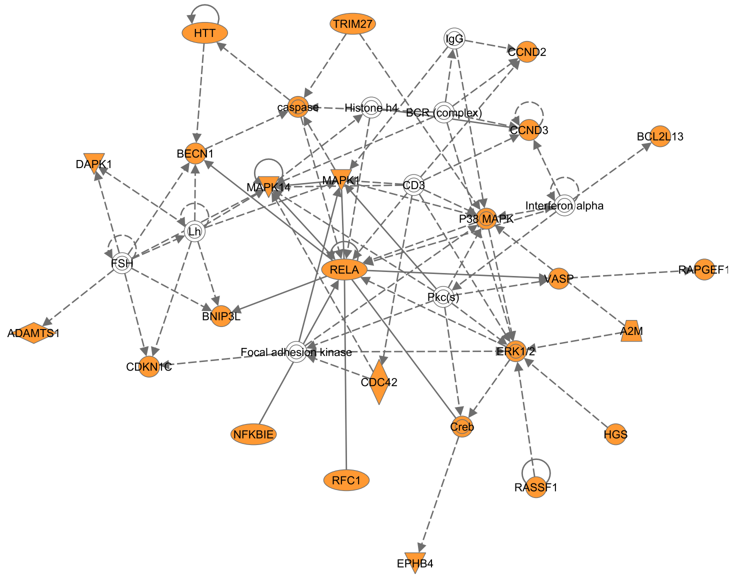

(k)

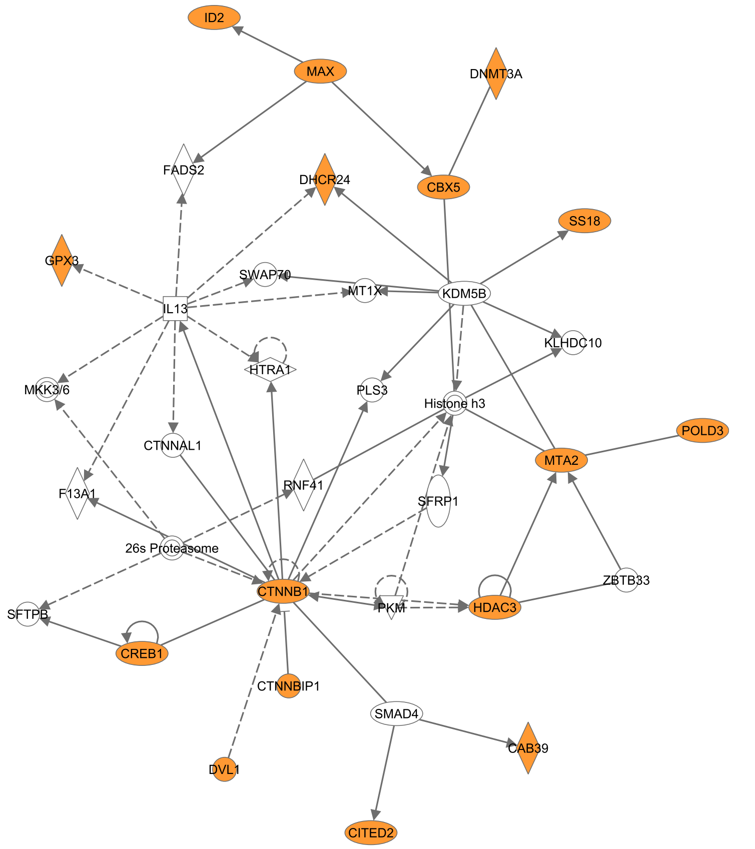

(l)

v) miRNA and RNAsnp overlap in all genes

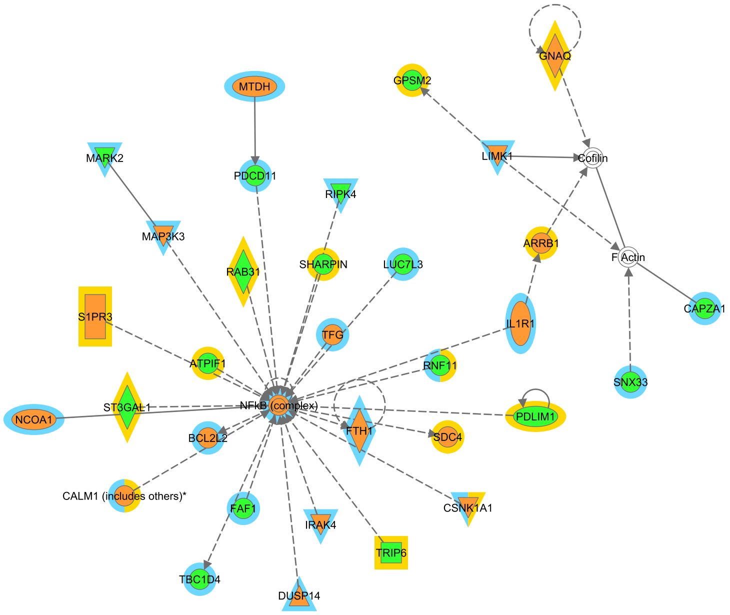

(m)

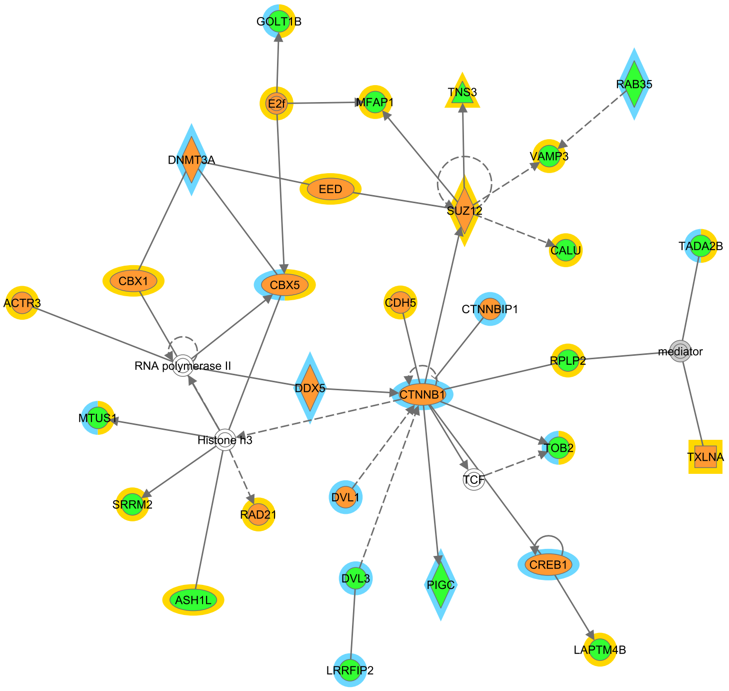

(n)

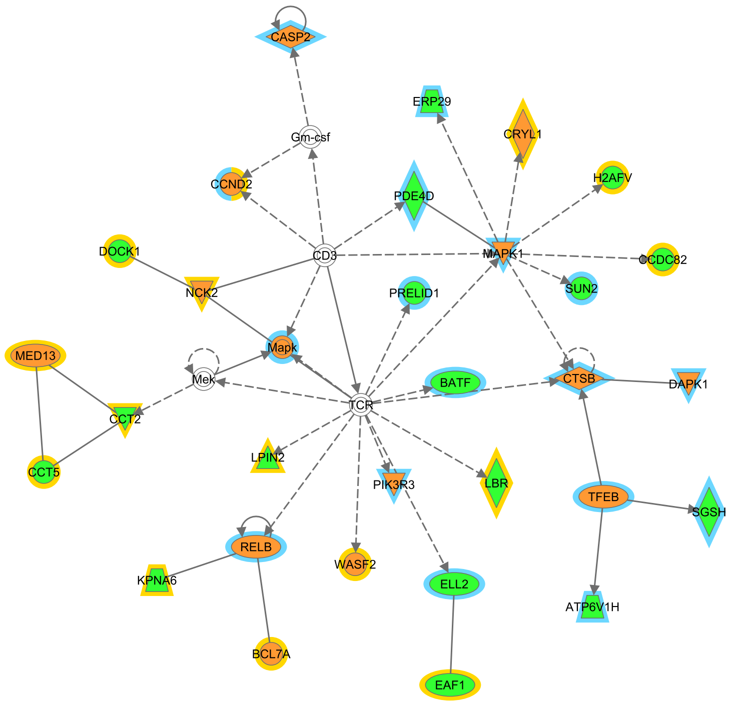

(o)

vi) miRNA and RNAsnp overlap in cancer releated genes

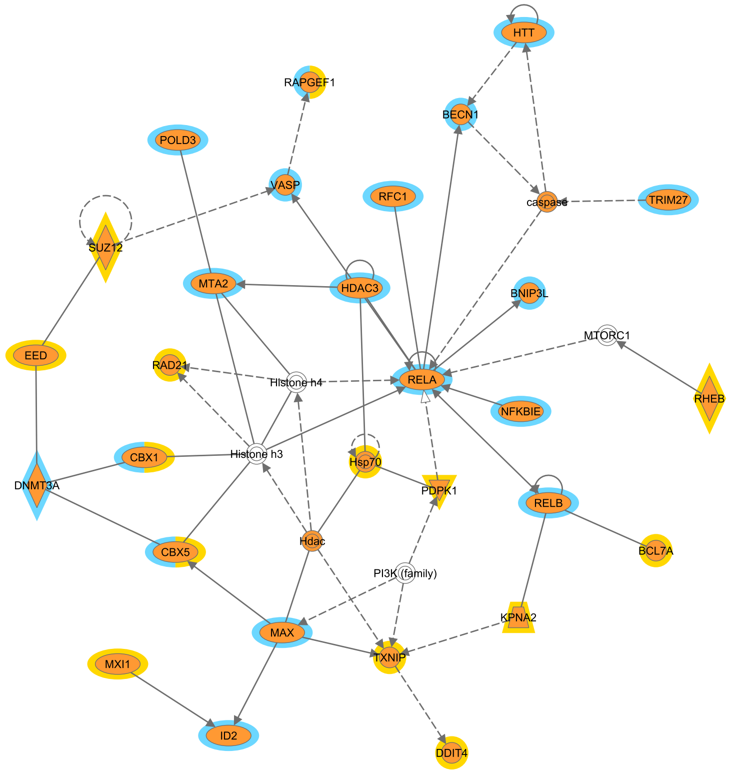

(p)

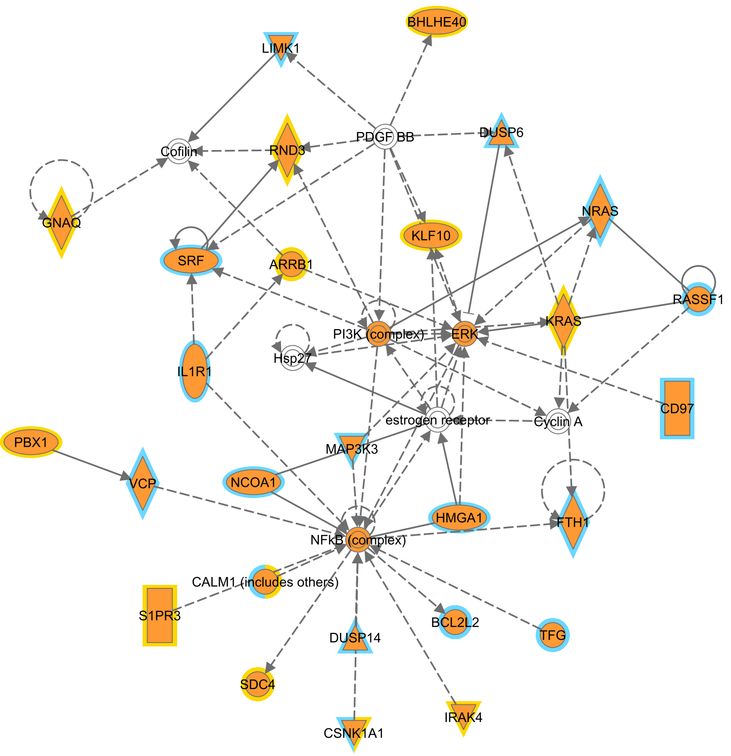

(q)

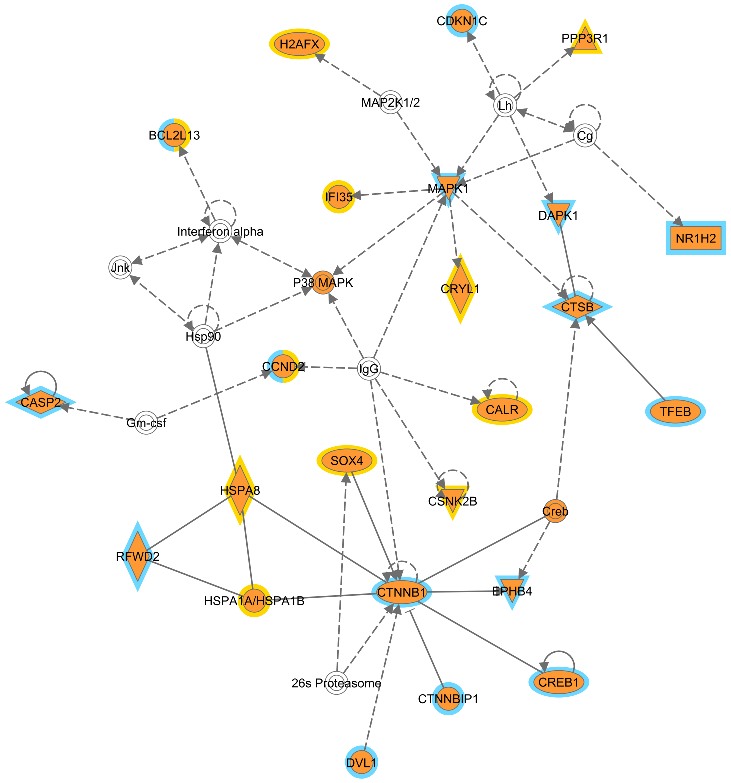

(r)

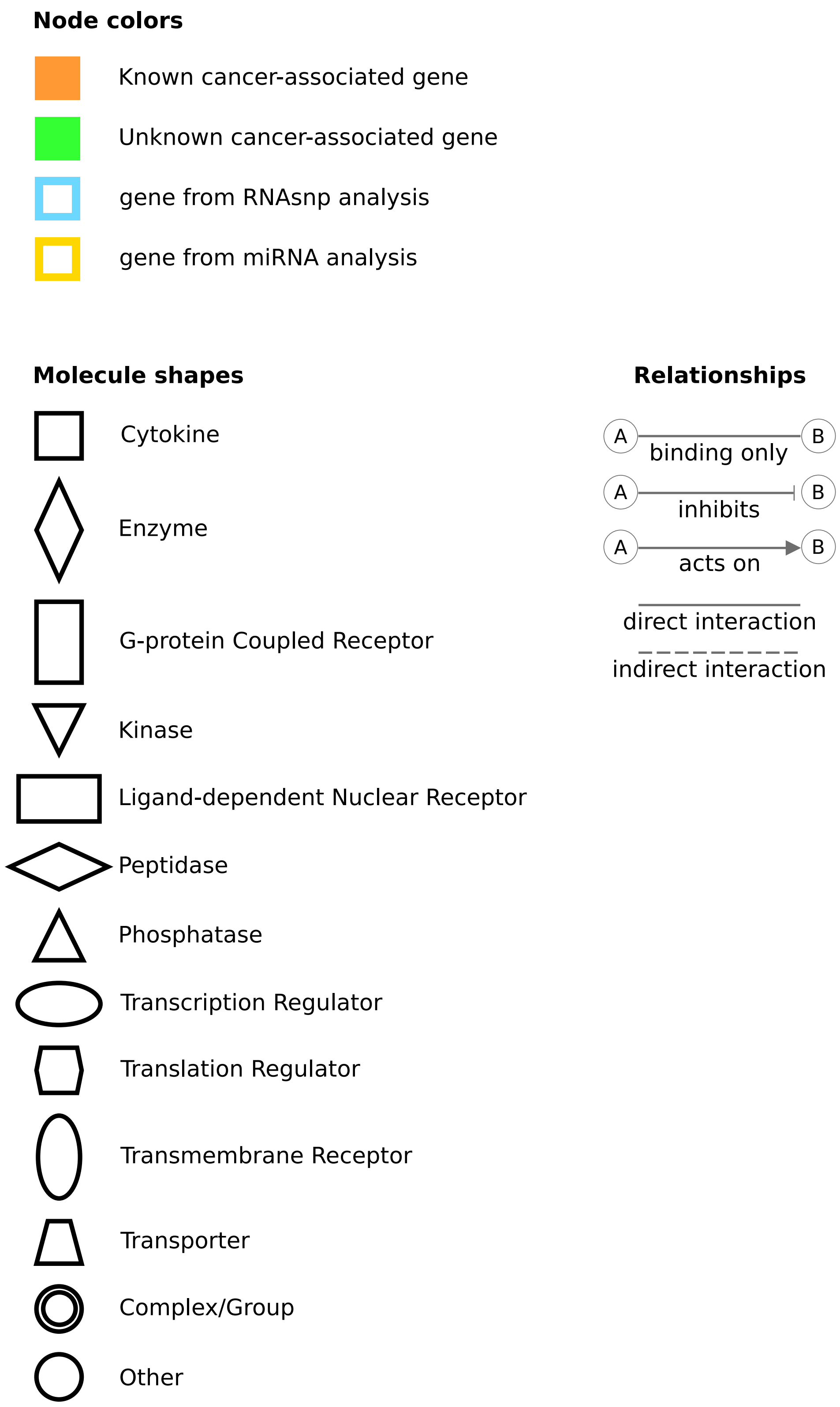

Figure S4: The networks represent the interaction between genes that were predicted to have disruptive SNVs obtained from miRNA analysis (a to f), RNAsnp analysis (g to l) and combination of both (m to r). The molecular function corresponds to each network is reported in Table 7. The gene nodes were colored to differentiate the known (orange) and unknown (green) cancer-associated genes, and the color outside the node indicates whether the gene comes from miRNA (yellow) or RNAsnp (blue) or both.
