# Supplementary material for: Transcriptome-Wide Analysis of UTRs in Non-Small Cell Lung Cancer Reveals Cancer-Related Genes with SNV-Induced Changes on RNA Secondary Structure and miRNA Target Sites
Source: PLoS One. 2014 Jan 8;9(1):e82699. doi: 10.1371/journal.pone.0082699 (PMC3885406; doi:10.1371/journal.pone.0082699)
Supplement: Table S1 — Filtration steps in the miRNA pipeline tested on known examples. (PDF) [file pone.0082699.s009.pdf]

SNP ID	dbSNP	RefSeq	SNP	miRNA ID	ΔG_{WT}	ΔG_{SNP}	*lr*	TargetScan seed change	$\|lr\| >$ 0.276	Ago CLIP-Seq
SNP01[1]	rs12720208	NM_019851	C182T	hsa-miR-433	-11.30	-8.83	-0.356	*	*	
				hsa-miR-148a-3p	-20.47	-26.52	0.374	*	*	
SNP02[2]	rs1063320	NM_002127	C590G	hsa-miR-148b-3p	-20.83	-26.88	0.368	*	*	
				hsa-miR-152	-16.35	-22.40	0.454	*	*	
SNP03[3]	rs56109847	NM_001256613	G76A	hsa-miR-510	-23.69	-17.35	-0.449	*	*	
SNP04[4]	rs5186	NM_000685	A86C	hsa-miR-155-5p	-13.52	-10.07	-0.425	*	*	
SNP05[5]	-	NM_052910	G689A	hsa-miR-24-1-5p	-20.00	-20.65	0.046	*		
SNP06[6]	rs1799782	NM_006297	C700T	hsa-miR-138-5p	-16.74	-16.61	-0.011			
SNP07[6]	rs1800470	NM_000660	C911T	hsa-miR-187-3p	-20.57	-15.49	-0.409		*	
SNP08[7]	rs712	NM_004985	T2505G	hsa-let-7b-5p	-19.71	-19.52	-0.014			*
				hsa-let-7g-5p	-8.47	-12.00	0.503		*	*
				hsa-let-7d-5p	-7.86	-16.97	1.110		*	*
				hsa-let-7a-5p	-14.91	-17.62	0.241			*
SNP09[8]	rs8126	NM_006291	C1872T	hsa-miR-184	-18.27	-15.47	-0.240	*		
SNP10[9]	rs1044129	NM_001036	A839G	hsa-miR-367-3p	-9.88	-7.96	-0.312		*	
SNP11[10]	rs17084733	NM_000222	G3235A	hsa-miR-221-3p	-20.86	-17.20	-0.278		*	
SNP12[11]	rs11145043	NM_181425	G1659U	hsa-miR-124-3p	-10.86	-15.58	0.521	*	*	
SNP13[12]	rs1434536	NM_001256794	C1804U	hsa-miR-125b-5p	-17.45	-14.75	-0.243	*		
SNP14[13]	rs465646	NM_002912	T10177C	hsa-miR-32-5p	-16.78	-18.71	0.157			
				hsa-miR-25-3p	-19.67	-19.67	0.000			
SNP15[14]	rs4143815	NM_014143	G1376C	hsa-miR-570-3p	-5.86	—	DESTROY	*	N/A	
SNP16[15]	rs3218073	NM_001238	C1748T	hsa-miR-151a-5p	-21.42	-19.60	-0.128	*		
SNP17[16]	rs2239680	NM_001168	T698C	hsa-miR-335-5p	-14.91	-10.98	-0.441	*	*	*
SNP18[17]	rs4245739	NM_001204172	A693C	hsa-miR-191-5p	—	-11.75	CREATE	*	N/A	
SNP19[18]	rs12537	NM_153050	C5511U	hsa-miR-181a-5p	-10.85	-15.76	0.539	*	*	

Table S1: Filtration steps in the miRNA pipeline tested on known examples. Columns 1–5 list the internal SNP identifier, dbSNP and RefSeq accessions, information of the actual polymorphism, and the miRNA (ID adjusted to miRBase V19) whose binding is shown to be affected. Columns 6–8 hold the binding energies as predicted by miRanda for WT and SNP variant respectively, and the log ratio of the two for those classified as *alter*, "DESTROY" or "CREATE" otherwise. The last three columns indicate with a '*' whether that pair gets through the respective filter. Of the 19 SNPs (25 interactions) that were extracted from the literature, TargetScan found seed match changes for 13 SNVs in 15 interactions. For SNP10 and SNP15 no interaction passes the -11 kcal/mol threshold. The *lr* for 14 out of 23 interactions in the *alter* set is higher than our threshold. Only two SNPs are found inside Ago CLIP-Seq peak clusters with BC ≥ 2.

References

[1] G. Wang, J. M. van der Walt, G. Mayhew, Y.-J. Li, S. Züchner, W. K. Scott, E. R. Martin, and J. M. Vance, "Variation in the mirna-433 binding site of fgf20 confers risk for parkinson disease by overexpression of alpha-synuclein.," *Am J Hum Genet*, vol. 82, pp. 283–289, Feb 2008.

[2] Z. Tan, G. Randall, J. Fan, B. Camoretti-Mercado, R. Brockman-Schneider, L. Pan, J. Solway, J. E. Gern, R. F. Lemanske, D. Nicolae, and C. Ober, "Allele-specific targeting of micrornas to hla-g and risk of asthma.," *Am J Hum Genet*, vol. 81, pp. 829–834, Oct 2007.

[3] J. Kapeller, L. A. Houghton, H. Mönnikes, J. Walstab, D. Möller, H. Bönisch, B. Burwinkel, F. Autschbach, B. Funke, F. Lasitschka, N. Gassler, C. Fischer, P. J. Whorwell, W. Atkinson, C. Fell, K. J. Büchner, M. Schmidtmann, I. van der Voort, A.-S. Wisser, T. Berg, G. Rappold, and B. Niesler, "First evidence for an association of a functional variant in the microrna-510 target site of the serotonin receptor-type 3e gene with diarrhea predominant irritable bowel syndrome.," *Hum Mol Genet*, vol. 17, pp. 2967–2977, Oct 2008.

[4] P. Sethupathy, C. Borel, M. Gagnebin, G. R. Grant, S. Deutsch, T. S. Elton, A. G. Hatzigeorgiou, and S. E. Antonarakis, "Human microrna-155 on chromosome 21 differentially interacts with its polymorphic target in the agtr1 3' untranslated region: a mechanism for functional single-nucleotide polymorphisms related to phenotypes.," *Am J Hum Genet*, vol. 81, pp. 405–413, Aug 2007.

[5] J. F. Abelson, K. Y. Kwan, B. J. O'Roak, D. Y. Baek, A. A. Stillman, T. M. Morgan, C. A. Mathews, D. L. Pauls, M.-R. Rasin, M. Gunel, N. R. Davis, A. G. Ercan-Sencicek, D. H. Guez, J. A. Spertus, J. F. Leckman, L. S. Dure, R. Kurlan, H. S. Singer, D. L. Gilbert, A. Farhi, A. Louvi, R. P. Lifton, N. Sestan, and M. W. State, "Sequence variants in slitrk1 are associated with tourette's syndrome.," *Science*, vol. 310, pp. 317–320, Oct 2005.

[6] M. S. Nicoloso, H. Sun, R. Spizzo, H. Kim, P. Wickramasinghe, M. Shimizu, S. E. Wojcik, J. Ferdin, T. Kunej, L. Xiao, S. Manoukian, G. Secreto, F. Ravagnani, X. Wang, P. Radice, C. M. Croce, R. V. Davuluri, and G. A. Calin, "Single-nucleotide polymorphisms inside microrna target sites influence tumor susceptibility.," *Cancer Res*, vol. 70, pp. 2789–2798, Apr 2010.

[7] L. J. Chin, E. Ratner, S. Leng, R. Zhai, S. Nallur, I. Babar, R.-U. Muller, E. Straka, L. Su, E. A. Burki, R. E. Crowell, R. Patel, T. Kulkarni, R. Homer, D. Zelterman, K. K. Kidd, Y. Zhu, D. C. Christiani, S. A. Belinsky, F. J. Slack, and J. B. Weidhaas, "A snp in a let-7 microrna complementary site in the kras 3' untranslated region increases non-small cell lung cancer risk.," *Cancer Res*, vol. 68, pp. 8535–8540, Oct 2008.

[8] Z. Liu, S. Wei, H. Ma, M. Zhao, J. N. Myers, R. S. Weber, E. M. Sturgis, and Q. Wei, "A functional variant at the mir-184 binding site in tnfaip2 and risk of squamous cell carcinoma of the head and neck.," *Carcinogenesis*, vol. 32, pp. 1668–1674, Nov 2011.

[9] L. Zhang, Y. Liu, F. Song, H. Zheng, L. Hu, H. Lu, P. Liu, X. Hao, W. Zhang, and K. Chen, "Functional snp in the microrna-367 binding site in the 3'utr of the calcium channel ryanodine receptor gene 3 (ryr3) affects breast cancer risk and calcification.," *Proc Natl Acad Sci U S A*, vol. 108, pp. 13653–13658, Aug 2011.

[10] S. E. Godshalk, T. Paranjape, S. Nallur, W. Speed, E. Chan, A. M. Molinaro, A. Bacchiocchi, K. Hoyt, K. Tworkoski, D. F. Stern, M. Sznol, S. Ariyan, R. Lazova, R. Halaban, K. K. Kidd, J. B. Weidhaas, and F. J. Slack, "A variant in a microrna complementary site in the 3' utr of the kit oncogene increases risk of acral melanoma.," *Oncogene*, vol. 30, pp. 1542–1550, Mar 2011.

[11] S. Bandiera, F. Cartault, A.-S. Jannot, E. Hatem, M. Girard, L. Rifai, C. Loiseau, A. Munnich, S. Lyonnet, and A. Henrion-Caude, "Genetic variations creating microrna target sites in the fxn 3'-utr affect frataxin expression in friedreich ataxia.," *PLoS One*, vol. 8, no. 1, p. e54791, 2013.

[12] P. Saetrom, J. Biesinger, S. M. Li, D. Smith, L. F. Thomas, K. Majzoub, G. E. Rivas, J. Alluin, J. J. Rossi, T. G. Krontiris, J. Weitzel, M. B. Daly, A. B. Benson, J. M. Kirkwood, P. J. O'Dwyer, R. Sutphen, J. A. Stewart, D. Johnson, and G. P. Larson, "A risk variant in an mir-125b binding

site in bmpr1b is associated with breast cancer pathogenesis.," *Cancer Res*, vol. 69, pp. 7459–7465, Sep 2009.

[13] S. Zhang, H. Chen, X. Zhao, J. Cao, J. Tong, J. Lu, W. Wu, H. Shen, Q. Wei, and D. Lu, "Rev3l 3'utr 460 t¿c polymorphism in microrna target sites contributes to lung cancer susceptibility.," *Oncogene*, vol. 32, pp. 242–250, Jan 2013.

[14] W. Wang, F. Li, Y. Mao, H. Zhou, J. Sun, R. Li, C. Liu, W. Chen, D. Hua, and X. Zhang, "A mir-570 binding site polymorphism in the b7-h1 gene is associated with the risk of gastric adenocarcinoma.," *Hum Genet*, vol. 132, pp. 641–648, Jun 2013.

[15] Y. Liu, H. Cai, J. Liu, H. Fan, Z. Wang, Q. Wang, M. Shao, X. Sun, J. Diao, Y. Liu, Y. Shi, and Q. Fan, "A mir-151 binding site polymorphism in the 3'-untranslated region of the cyclin e1 gene associated with nasopharyngeal carcinoma.," *Biochem Biophys Res Commun*, vol. 432, pp. 660–665, Mar 2013.

[16] Y. Zu, J. Ban, Z. Xia, J. Wang, Y. Cai, W. Ping, and W. Sun, "Genetic variation in a mir-335 binding site in birc5 alters susceptibility to lung cancer in chinese han populations.," *Biochem Biophys Res Commun*, vol. 430, pp. 529–534, Jan 2013.

[17] J. Wynendaele, A. Böhnke, E. Leucci, S. J. Nielsen, I. Lambertz, S. Hammer, N. Sbrzesny, D. Kubitza, A. Wolf, E. Gradhand, K. Balschun, I. Braicu, J. Sehouli, S. Darb-Esfahani, C. Denkert, C. Thomssen, S. Hauptmann, A. Lund, J.-C. Marine, and F. Bartel, "An illegitimate microrna target site within the 3' utr of mdm4 affects ovarian cancer progression and chemosensitivity.," *Cancer Res*, vol. 70, pp. 9641–9649, Dec 2010.

[18] Y. Lin, Y. Nie, J. Zhao, X. Chen, M. Ye, Y. Li, Y. Du, J. Cao, B. Shen, and Y. Li, "Genetic polymorphism at mir-181a binding site contributes to gastric cancer susceptibility.," *Carcinogenesis*, vol. 33, pp. 2377–2383, Dec 2012.
